# Crosstalk of three novel types of programmed cell death defines distinct microenvironment characterization and pharmacogenomic landscape in breast cancer

**DOI:** 10.3389/fimmu.2022.942765

**Published:** 2022-08-11

**Authors:** Lijun Xu

**Affiliations:** Renji Hospital, School of Medicine, Shanghai Jiao Tong University, Shanghai, China

**Keywords:** breast cancer, CD_Score, immunotherapy, programmed cell death, tumor microenvironment, tumor mutation burden

## Abstract

**Background:**

Prior studies have highlighted that novel programmed cell death (PCD) modalities, including ferroptosis, pyroptosis, and necroptosis, are correlated with tumor progression and antitumor immunity. Nonetheless, comprehensive analysis of tumor microenvironment (TME) profiles mediated by the crosstalk of distinct PCD forms has not been conducted in breast cancer (BC).

**Methods:**

Here, we curated 34 identified PCD-associated genes (PCDAGs) and applied the consensus clustering algorithm to establish PCD-mediated tumor patterns in BC. Subsequently, based on prognostic differentially expressed genes extracted from distinct PCD-mediated patterns, we applied the LASSO algorithm to construct CD_Score. Furthermore, the correlation analysis between CD_Score and TME features, molecular subtypes, clinicopathological characteristics, drug response, and immunotherapeutic efficacy was performed.

**Results:**

Three distinct PCD-clusters were determined among 2,038 BC samples, which did not only display different clinical outcomes but highly correlated to the established immunological tumor phenotypes: “desert,” “excluded,” and “inflamed” immune profiles. Based on the CD_Score derived from the PCD-related gene signature, BC patients could be stratified into CD_Score-low and -high group, of which the former displayed satisfactory survival outcome and enhanced immune infiltration. Further exploration identified that the CD_Score-high group significantly correlated with elevated neoantigen load and higher mutation frequency in SMGs (e.g., TP53 and MAP3K1) and reduced expression of immune checkpoint proteins.

**Conclusions:**

This research is the first to emphasize the close relationship between distinct cell death modalities and the diversity and complexity of immune infiltration in TME. We established the CD_Score, which could help enhance our cognition of TME features and facilitate the clinical application of immunotherapy.

## Introduction

Globally, breast cancer (BC) is the most frequently occurring cancer and the leading cause of cancer-related mortality among female populations (1, 2). According to global cancer statistics in 2020, there were an estimated 2.3 million newly diagnosed BC patients, accounting for 11.7% of all cancer cases and nearly 685,000 related deaths (3). As a new treatment modality, immunotherapy with immune checkpoint inhibitor (ICI) monoclonal antibodies has elicited durable objective responses and substantially prolonged overall survival (OS) for BC patients (4, 5). Despite the impressive impact of immunotherapy, a substantial proportion of BC patients remain unresponsive to these agents (6), because resistance to apoptosis is an essential hallmark of cancer (7, 8). Thus, the exploration of novel cell death modalities that could bypass apoptosis has gradually emerged as a promising strategy for cancer treatment.

As a newly discovered form of programmed cell death (PCD) characterized by iron dependency and lipid peroxide accumulation (9, 10), ferroptosis is precisely regulated by multiple regulators, which could either trigger or suppress this PCD process. Recently, accumulating evidence has highlighted that the selective induction of ferroptosis could effectively suppress tumor growth, even for chemotherapy-resistant tumor cells (11, 12). Pyroptosis represents an emerging proinflammatory type of PCD characterized by cytoplasmic membrane pore formation, cell swelling, and rapid lysis (13–15). This rupture contributes to the secretion of intracellular proinflammatory contents, which are involved in the initiation and progression of cancer. Necroptosis is caspase-independent necrotic cell death that occurs downstream of PRK1 and RIPK3, which could form a super-molecular complex called the necrosome to activate necroptosis (16–18). Necroptotic cells could permeabilize plasma membranes. expel intracellular components and further trigger the initiation of immune response, demonstrating that necroptosis is intimately associated with oncogenesis and cancer progression.

Recently, as much progress has been made in deepening our knowledge of the heterogeneous and complex properties of tumor microenvironment (TME) (composed of tumor and immune cells, cancer-associated fibroblasts and endothelial cells, and secreted factors), the involvement of TME in cancer initiation and progression has been gradually recognized (19–21). For example, the characterization of the proportion of CD3^+^ and CD8^+^ T lymphocyte subpopulations infiltrating the tumor center and margin plays an indispensable role in predicting tumor recurrence and mortality in BC (22). Besides, immunotherapies, particularly agents targeting ICI: PD-L1 and CTLA-4, have elicited durable antitumor effects for BC patients (5, 6). Assessment of TME-infiltrating characteristics has led to remarkable success in the prediction of therapeutic efficacy and the development of novel immunotherapeutic approaches (23–25). Thus, identification of immune subtypes by comprehensively parsing the TME components will help predict and direct immunotherapeutic strategies.

Previously, investigators have identified a significant correlation between novel PCD modalities and TME-infiltrating immune cells in people with cancer. For example, Wang et al. discovered that CD8^+^ T cells activated by cancer immunotherapy could release IFNγ to reduce SLC3A2 and SLC7A11 expression, leading to cell lipid peroxidation and ferroptotic cell death in cancer cells (26). Besides, in melanoma, the combination of BRAF and MEK inhibitors could promote the pyroptosis protein GSDME cleavage and HMGB1 release. GSEME-deficient melanoma exhibited defective HMGB1 release, decreased T cells and elevated dendritic cell infiltration (27). However, due to the technical limitations, most of the current studies have focused on the role of individual cell death in TME. A comprehensive analysis of the correlation between the crosstalk of novel PCD modalities and immune microenvironment has not been conducted.

In this research, we comprehensively evaluated the correlation between the crosstalk of ferroptosis, necroptosis, and pyroptosis and infiltrating immune cells, by integrating the genomic and transcriptomic features from 2,038 BC samples curated from the GEO and TCGA databases. Three distinct PCD-mediated patterns were established using the unsupervised consensus clustering algorithm, which was highly consistent with the previously established immunological tumor phenotypes: “excluded”, “inflamed”, and “desert” immune profiles, highlighting the indispensable role of PCD in the regulation of TME characteristics. Moreover, based on the prognostic PCD-related gene signature, we established the CD_Score, which could predict the therapeutic efficacy of chemotherapeutic drugs and immunotherapies, highlighting that PCD played a vital role in directing therapeutic interventions for BC.

## Materials and methods

### Data acquisition

We collected gene expression profiles of BC patients and the corresponding clinical features from two publicly available databases: TCGA (https://portal.gdc.cancer.gov/) and GEO (https://www.ncbi.nlm.nih.gov/geo/). The selection criteria for BC datasets were adapted from the workflow of Xu et al. (28), and a total of 2,038 BC samples were enrolled in this research, including those from the TCGA-BRCA (N = 1,091), GSE20685 (N = 327) (29), GSE16446 (N = 120) (30), GSE88770 (N = 117) (31), GSE58812 (N = 107) (32), GSE42568 (N = 104) (33), GSE20711 (N = 88) (34), and GSE135565 (N = 84) (35) datasets (**Table S1**). As for the TCGA-BRCA dataset, we downloaded FPKM value profiles and transformed them into the TPM format, which was more similar to microarray data and more comparable between samples. The “Homo_sapiens.GRCh38.104.chr.gtf” curated from the ENSEMBLE website was used as an annotation file to map the ensemble ID to gene symbol. Since these GSE datasets shared the same microarray platform, the annotation file of GPL570 was downloaded and used to map the probes. While merging the expression matrix of these eight BC datasets into one meta-cohort, we applied the “ComBat” function from the R “SVA” package to correct the batch effect (36). The genomic mutation data of the TCGA-BRCA obtained from the UCSC Xena database (http://xena.ucsc.edu/) was used for copy number variation (CNV) and somatic mutation analysis. The tumor mutation burden (TMB) was calculated based on the total number of nonsynonymous somatic mutations, which included splice site mutations, inflame mutations, frameshift mutations, nonsense mutations, and missense mutations. The localization of the CNV landscape of PCD-associated genes (PCDAGs) on the human chromosome was illustrated using the R “Rcircos” package (37).

### Consensus clustering analysis of PCDAGs

A total of 34 PCDAGs were procured from the GeneCards (https://www.genecards.org/) database (38), which provided comprehensive knowledge of all human annotated and predicted genes. Detailed information on these PCDAGs was presented in **Table S2**. According to the expression of the PCDAGs, we performed consensus clustering analysis (39) and stratified BC patients into distinct PCD-mediated tumor patterns. To select the optimal proportion of cluster and guarantee its stability, we performed such analysis based on the following stratification criteria: (1) each group consisted of an adequate sample size; (2) the curve of the cumulative distribution function declined at a gradual and smooth level; and (3) when the clustering was completed, the intragroup relationship increased, while the intergroup relationship decreased.

### Gene set variation analysis (GSVA) and evaluation of TME characteristics

To compare the differences in biological behavior among distinct PCD-mediated patterns, we used GSVA with the R package “GSVA” (40) and regarded adjusted P-value <0.05 as the filtering criterion. The gene sets “c2.cp.kegg.v7.5.1.symbols.gmt” and “h.all.v7.5.1.symbols.gmt” obtained from the MSigDB database were used as the well-defined biological signatures (41). To explore the relationship between the PCD-mediated patterns and TME landscape, we applied two validated algorithms: single-sample gene set enrichment analysis (ssGSEA) and CIBERSORT, both of which could quantify the difference in the proportion of TME immune cells. As to the ssGSEA algorithm, we estimated the bio-similarity of TME-infiltrating immune cells based on the multidimensional scaling and Gaussian fitting model. To define the relative abundance of immune cells in each sample, we quantified the enrichment score of TME infiltrating cells and normalized the score to a unity distribution from 0 to 1. The deconvolution approach CIBERSORT could determine the relative expression of immune cells in each tumor based on the expression profile of 547 reference genes (42). Moreover, to estimate the level of infiltrating stromal and immune components, we used the R “estimate” package with default parameters and applied the Estimation of Stromal and Immune Cells in Malignant Tumors using Expression Data (ESTIMATE) algorithm to infer tumor purity (43).

### Identification of differentially expressed genes (DEGs) among distinct PCD-mediated patterns and exploration of their functional annotation

The consensus clustering analysis has satisfactorily stratified BC patients into distinct PCD-mediated patterns. Subsequently, we applied the R “limma” package (44) and employed its empirical Bayesian approach to compare the gene expression value between distinct patterns and identified DEGs with the significance criteria of adjusted P-value <0.001. Besides, the R “clusterprofiler” package (45) was applied to execute the functional enrichment analyses of DEGs and discover their potential functions and enriched pathways.

### Construction and validation of the CD_Score scoring system

To establish a predictive model by which clinicians could estimate the likelihood of survival of BC patients, we first employed the above overlapping DEGs into a univariate Cox regression analysis and extracted those OS-related ones. Then, according to the expression profiles of prognostic DEGs, the unsupervised clustering algorithm classified BC patients into distinct subtypes for deeper analysis. Finally, all the BC patients were randomly assigned (ratio = 1:1) to either the discovery or validation dataset. In the discovery dataset, the “glmnet” R package was used to perform LASSO regression analysis, which was capable of achieving the risk minimization of overfitting, analyzing the change trajectory of prognostic DEGs, and performing 10-fold validation to determine the optimal value of penalty parameter and candidate genes (46). Based on the candidate genes, we performed multivariate Cox analysis to establish a PCD-related prognostic model and defined it as CD_Score (“Cell Death_Score”), whose formula was calculated as follows: risk score = ∑ (β_K_ ∗ G_K_), where G_K_ and β_K_ represented the normalized expression value and coefficient of gene _K_, respectively.

According to the median risk score, BC patients in the discovery dataset were separated into CD_Score-low and -high group and then subjected to Kaplan–Meier survival curve analysis. Similarly, patients in the validation dataset were also divided into CD_Score-high and -low group for the subsequent ROC and Kaplan–Meier analysis.

### Correlation of the CD_Score with tumor mutational landscape, immunotherapeutic efficacy, and drug susceptibility

Significantly mutated genes (SMGs) (47, 48) between different CD_Score groups were recognized using the “MutSigCV” algorithm, which could address mutation frequency in a mutational context-specific manner and further determine genes significantly enriched in non-silent somatic mutations. We applied the R “maftools” package (49) and illustrated the waterfall plot to identify the mutational profiles of PCDAGs and SMGs in the TCGA-BRCA dataset.

The immunophenoscore (IPS) (50) of BC patients was curated from the TCIA database (https://tcia.at/home). Based on the machine learning methods, IPS was evaluated without bias and presented on a scale of 0–10. This procedure was carried out based on the immunogenicity-determining genes in four representative cell types: effector cells, immunosuppressive cells, immunomodulators, and MHC molecules. Generally, higher IPS score represented the elevated immunogenicity and active immunotherapeutic responsiveness.

Individual chemotherapeutic responses were evaluated on the basis of Genomics of Drug Sensitivity in Cancer (51), the public pharmacogenomic database that qualifies users to predict the sensitivity of 138 commonly used chemotherapeutic drugs. Through the establishment of the ridge regression model based on the gene expression spectrum of the TCGA-BRCA dataset and GDSC cell line, we applied the R “pRRophetic” package (52) to measure the half inhibitory concentration (IC_50_) (53).

## Results

### Genomic and transcriptomic profilings of PCDAGs in BC

**Figure 1:**
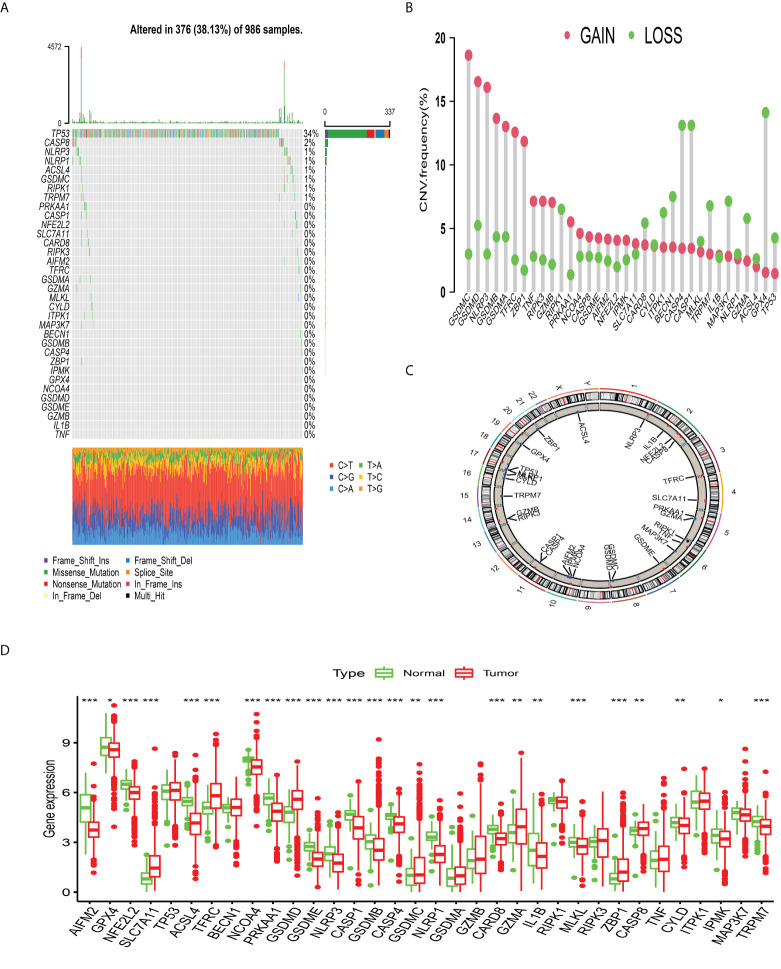
The landscape of genetic alterations of programmed cell death-associated genes in breast cancer**. (A)** 376 of the 986 BC patients experienced genetic alterations of 34 PCDAGs, with a frequency of 38.13%, mostly including missense mutation, frame shift deletion and nonsense mutation. **(B)** The CNV mutation frequency of 34 PCDAGs was frequent. The column represented the alteration frequency. The amplification frequency, red dot; the deletion frequency, green dot. **(C)** The location of CNV alteration of PCDAGs on chromosomes. **(D)** The difference of mRNA expression levels of 34 PCDAGs between BC and normal samples. The asterisks represented the statistical P-value (*P <0.05; **P <0.01; ***P <0.001).

The enrichment analysis of PCDAGs *via* GO and Metascape was performed and we discovered that biological processes involved in ferroptosis, pyroptosis and necroptosis were highly overrepresented (**Figures S1A, B**). **Figure 1A** illustrates the incidence of somatic mutations in 34 PCDAGs on the TCGA-BRCA dataset. Out of 986 BRCA samples with available information of variant type and classification, 376 (38.13%) samples exhibited mutation in the PCDAGs, mainly including nonsense mutation, missense mutation and frame shift deletion. Taking into consideration the highest mutation frequency of TP53, we further compared the expression level of other PCDAGs and identified 26 differentially expressed PCDAGs among 666 TP53-wild and 314 TP53-mutant tumor samples (**Figure S2**). Besides, to compare the differences in biological processes between the PCDAG mutation and non-mutation group, we performed GSVA and discovered that cancer-related hallmark gene sets, including MTORC1 signaling pathway, hypoxia and IL6/JAK/STAT3 signaling pathway were strongly overrepresented in the mutation group (**Figure S1C**). As a pleiotropic cytokine involved in the regulation of inflammatory and immune response, IL6 performed a vital role in the JAK3/STAT3 pathway. Previous studies have demonstrated that IL6-mediated dysregulation of JAK/STAT3 pathway significantly correlates with proliferation, metastasis and survival of tumor cells. Based on these findings, we speculated that mutations in PCDAGs could lead to functional changes, thus influencing BC progression.

Subsequent exploration of 34 PCDAGs revealed a widespread frequency of CNV, among which GSDMC, GSDMD, and NLRP3 exhibited widespread CNV amplification, whose mean levels were 0.796, 0.74, and 0.681, respectively, while GPX4, CASP4, and CASP1 displayed prevalent heterozygous deletions (**Figure 1B**, **Table S3**). **Figure 1C** and **Table S4** visualized the location of CNV changes in PCDAGs on the chromosome. Besides, we investigated the transcriptional change of PCDAGs between BC and normal samples and discovered that PCDAGs with CNV amplification, such as GSDMC, GSDMD, and ZBP1, exhibited higher expression in BC samples compared with normal ones, while PCDAGs with CNV loss, such as GPX4, ACSL4, and NLRP1, displayed reduced expression in tumor ones (**Figures 1B, D**), demonstrating that CNV might participate in the regulation of PCDAG expression. However, some PCDAGs with amplified CNV demonstrated markedly decreased expression in BC tissues, such as NLRP3, GSDMB, and PRKAA1, while other PCDAGs with either CNV amplification or deletion demonstrated no obvious difference between BC and normal tissues. Therefore, although CNV could contribute to the expression variation of PCDAGs between tumor and normal samples, it is not recognized as the only regulator of gene expression. Other contributors, such as m6A modification, DNA methylation, and non-coding RNA, could also control the gene expression. These findings highlight the highly heterogeneous genomic and transcriptomic profiles of PCDAGs, and these imbalanced characteristics of PCDAG expression take on a vital role in BC tumorigenesis and progression.

### Identification of distinct PCDAGs-mediated tumor patterns

**Figure 2:**
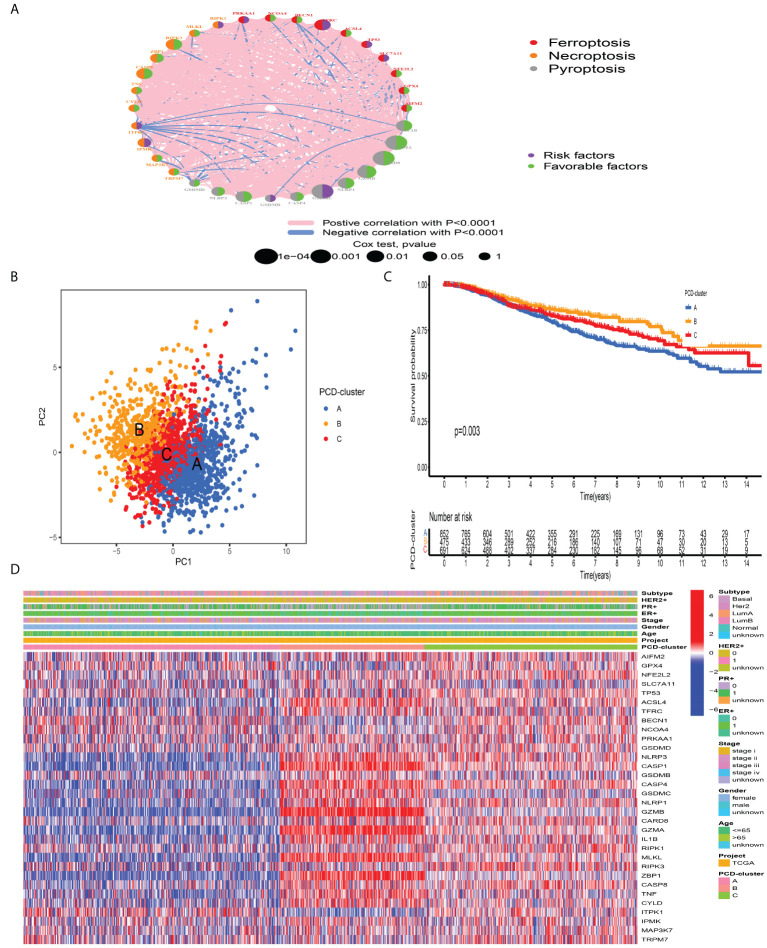
Tumor pattern clusters mediated by the programmed cell death-associated genes**. (A)** The interaction of expression on 34 PCDAGs in BC. The PCDAGs in three PCD-mediated tumor patterns were depicted by circles in different colors. Ferroptosis, red; Necroptosis, orange; Pyroptosis, gray. The lines connecting PCDAGs represented their interaction with each other. The size of each circle represented the prognostic effect of each regulator and scaled by P-value. Protective factors for patients’ survival were indicated by a green dot in the circle center and risk factors indicated by the purple dot in the circle center. **(B)** PCA of PCDAGs to distinguish between PCD-cluster A, B, and C **(C)** Kaplan–Meier curves of OS for 2,038 BC patients in the meta-cohort among different PCD-clusters. The numbers of patients in cluster A, B, and C were 859, 479, and 700 samples. **(D)** Unsupervised clustering of PCDAGs in one meta-cohort. The PCD-cluster, age, gender, ER, PR, HER2, molecular subtype, and clinical stage were used as patient annotations. Red represented the high expression of PCDAGs and blue represented low expression.

A total of 2,038 BC samples from eight datasets with available survival information (TCGA-BRCA, GSE20685, GSE16446, GSE88770, GSE58812, GSE42568, GSE20711, and GSE135565) were retained for subsequent analysis. Detailed characteristics of 2,038 BC samples are provided in **Table S1**. To define the prognostic significance of PCDAGs in BC, we applied the univariate Cox regression assay and Kaplan–Meier method and regarded P-value <0.05 as the filtering threshold (**Figure S3**, **Table S5**). As shown in **Figure 2A** and **Table S6**, this network illustrated the comprehensive features of the connections, interactions, and prognostic impact of PCDAGs in BC. The crosstalk among PCDAGs demonstrated that these three novel PCD modalities: ferroptosis, pyroptosis, and necroptosis, had a significant correlation with OS and played a crucial role in the establishment of PCDAGs-mediated patterns for BC patients.

According to the expression profiles of PCDAGs, the R “ConsensusClusterPlus” package was employed to quantitatively stratify BC patients with different PCD-mediated patterns. Through the consensus clustering analysis, we obtained three distinct patterns, including 859 samples in PCD-cluster A, 479 samples in cluster B, and 700 samples in cluster C (**Figure S4**). PCA demonstrated the obvious differences in the PCD transcription landscape among these three clusters (**Figure 2B**). The performance of these three PCD-clusters in predicting clinical outcome demonstrated that PCD-cluster B displayed a prominent survival benefit, followed by clusters C and A (**Figure 2C**). To further characterize and explore the clinical differences among distinct PCD-clusters, we focused on the TCGA-BRCA dataset, because it compromised the largest sample size and provided the most comprehensive patient information. **Figure 2D** presented the distribution of clinical features and PCDAGs expression profiling among PCD-clusters, and we discovered that PCDAGs, such as CASP1, CASP4, GZMB, and GZMA, were evidently elevated in PCD-cluster B.

### Characteristics of immune profile among distinct PCD-clusters

**Figure 3:**
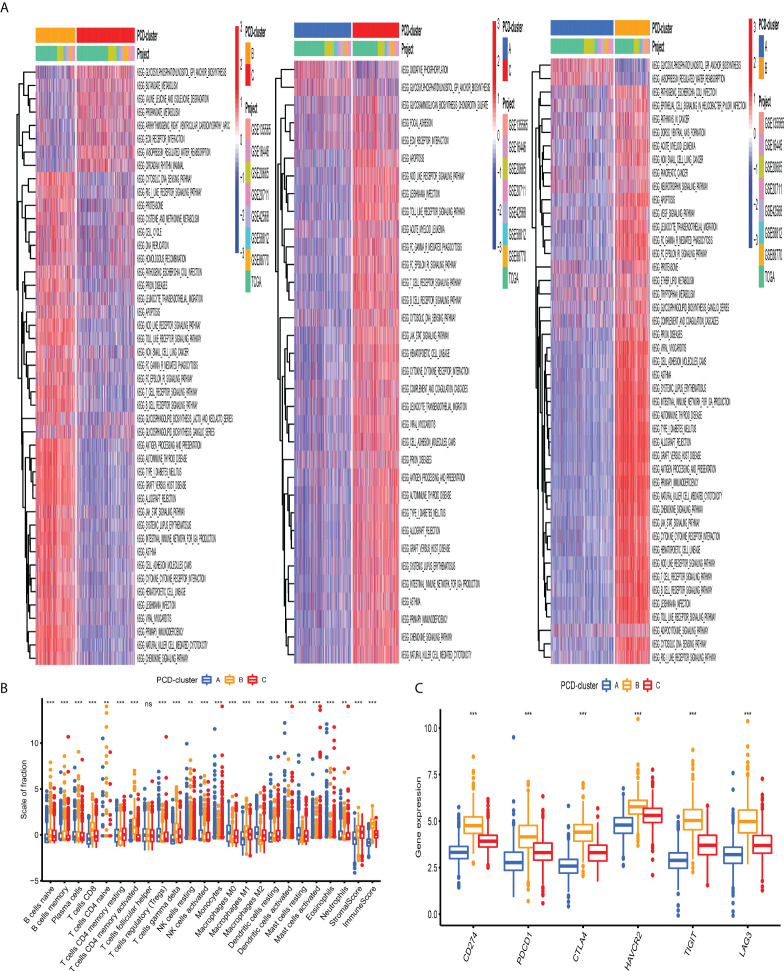
Biological behaviors and tumor microenvironment characteristics among distinct PCD-clusters**. (A)** Heatmap shows the GSVA score of representative KEGG pathways curated from MSigDB among distinct PCD-mediated tumor patterns. The meta-cohort compositions (GSE135565, GSE16446, GSE20685, GSE20711, GSE42568, GSE58812, GSE88770, and TCGA-BRCA) were used as sample annotations. **(B)** The fraction of antitumor immune cell subpopulations and immune score among distinct PCD-clusters using the CIBERSORT and ESTIMATE algorithms. Within each group, the scattered dots represented TME cell expression values. The thick line represented the median value. The bottom and top of the boxes were the 25th and 75th percentiles (interquartile range). The statistical difference of three PCD-clusters was compared through the Kruskal–Wallis H test. ns not significant; **P < 0.01; ***P < 0.001. **(C)** Comparison of the expression level of immunosuppressive molecules across distinct PCD-clusters.

To investigate the biological processes underling three PCD-mediated patterns, we performed GSVA and observed that PCD-cluster B with favorable prognosis was involved in immune activation pathways, such as T and B cell receptor signaling pathways, allograft rejection, and cytokine and cytokine receptor interaction (**Figure 3A**, **Table S7**). Besides, the expression level of eight immune activation-related transcripts was also elevated in PCD-cluster B (**Figure S5B**). PCD-cluster C was markedly enriched in stromal and carcinogenic activation pathways, including ECM receptor interaction, epithelial–mesenchymal transition, and TGF beta signaling pathway (**Figure S5A**, **Table S8**). To further characterize the TME features among distinct PCD-clusters, we conducted CIBERSORT to estimate the abundances of 23 TME immune cells (**Table S9**). As shown in **Figure 3B**, antitumor immune cell subpopulations, such as CD8^+^ T cells, M1 macrophages and activated memory CD4^+^ T cells, were strongly overrepresented in PCD-clusters B and C, while Treg cells and M2 macrophages were markedly elevated in cluster A. Furthermore, we used the ssGSEA algorithm to uncover the TME landscape (**Table S10**) and observed the same characteristics of antitumor lymphocyte subpopulations among these three PCD-clusters (**Figure S5C**). We also performed ESTIMATE analysis to assess the level of infiltrating stromal and immune cells among distinct PCD-clusters. Expectedly, we observed that PCD-clusters B and C displayed an elevated immune and stromal score, implying that cluster A had a higher tumor purity (**Figure 3B**). However, compared with PCD-cluster B, cluster C did not display an excellent prognosis. Previous studies demonstrated that tumors with an “excluded” immune profile displayed the presence of abundant immune cells. However, these immune cells were positioned in the stroma surrounding tumor cell nests rather than penetrating tumor parenchyma. The abundant stromal elements were considered T-cell suppressive. The results from GSVA analyses have demonstrated that PCD-cluster C displayed a significant correlation with stromal activation. Consequently, we speculated that stromal activation in PCD-cluster C inhibited the antitumor effect of immune cells. Subsequent analysis revealed that stromal activity was significantly enriched in PCD-cluster C, as some transcripts of the TGF beta/EMT pathway were strongly overrepresented (**Figure S5D**). Taking into consideration the role of ICI in predicting immunotherapeutic efficacy, we also compared the differences in ICI expression among distinct PCD-mediated patterns and observed a significantly upregulated expression in cluster B (**Figure 3C**). Taken together, we speculated that PCD-clusters corresponded to distinct tumor immunological phenotypes. PCD-cluster C was considered as an “excluded” immune profile, characterized by immune cell infiltration and stromal activation; cluster B as “inflamed” immune profile characterized by abundant infiltrating antitumor lymphocytes and immune activation, and cluster A as a “desert” immune profile, characterized by immune suppression.

### Construction of PCD-related gene signature

**Figure 4:**
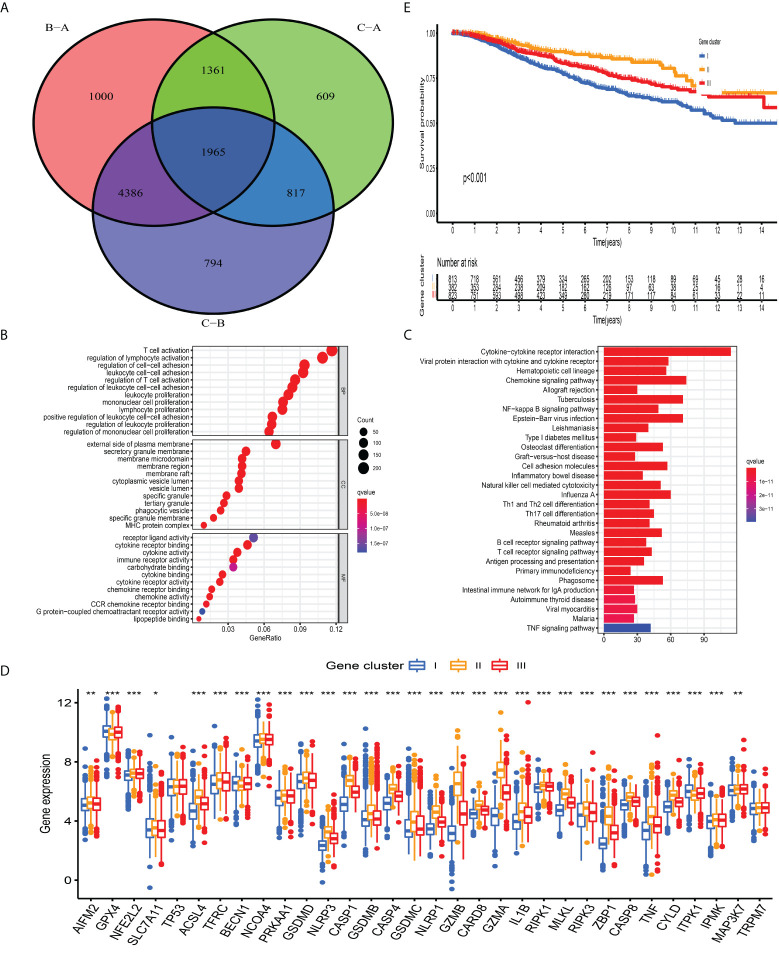
Construction of PCD-related gene signature and functional annotation**. (A)** A total of 1,965 DEGs among distinct PCD-clusters were shown in the Venn diagram. **(B, C)** Functional annotation for DEGs using GO and KEGG enrichment analyses. **(D)** The expression of 34 PCDAGs among three gene clusters. The upper and lower ends of the boxes represented an interquartile range of values. The lines in the boxes represented the median value and black dots showed outliers. The asterisks represented the statistical P-value (*P < 0.05; **P < 0.01; ***P < 0.001). The one-way ANOVA test was used to test the statistical differences among three gene clusters.

The previous unsupervised consensus clustering algorithm quantitatively classified BC samples into different PCD-mediated patterns. However, the genetic changes underlying these three clusters remain unclear. Thus, we introduced the R “limma” change to observe the change in the expression level of 16,436 genes among distinct PCD-clusters and obtained a total of 1,965 DEGs, which were termed as PCD-related gene signature (**Figure 4A**). GO annotation of DEGs revealed that biological processes implicated in immune activation were significantly enriched (**Figure 4B**). KEGG analysis also demonstrated that these DEGs performed a vital role in immune regulation (**Figure 4C**, **Table S11**), indicating that PCD was closely connected with TME. We then conducted univariate cox regression analysis to discover the prognostic impact of these 1,965 DEGs and determined 328 genes associated with OS for subsequent analysis (P-value <0.001, **Table S12**). Based on the 328 representative prognostic genes, we adopted the consensus clustering algorithm and determined three stable PCD-related transcriptome subtypes (**Figure S6**), which were termed PCD-gene clusters I, II, and III. As shown in **Figure 4D**, significant differences in the PCDAG expression level among distinct gene clusters were observed. Further prognostic investigation demonstrated that gene cluster II displayed the best prognosis, followed by gene clusters III and I (**Figure 4E**).

### Generation and validation of CD_Score

**Figure 5:**
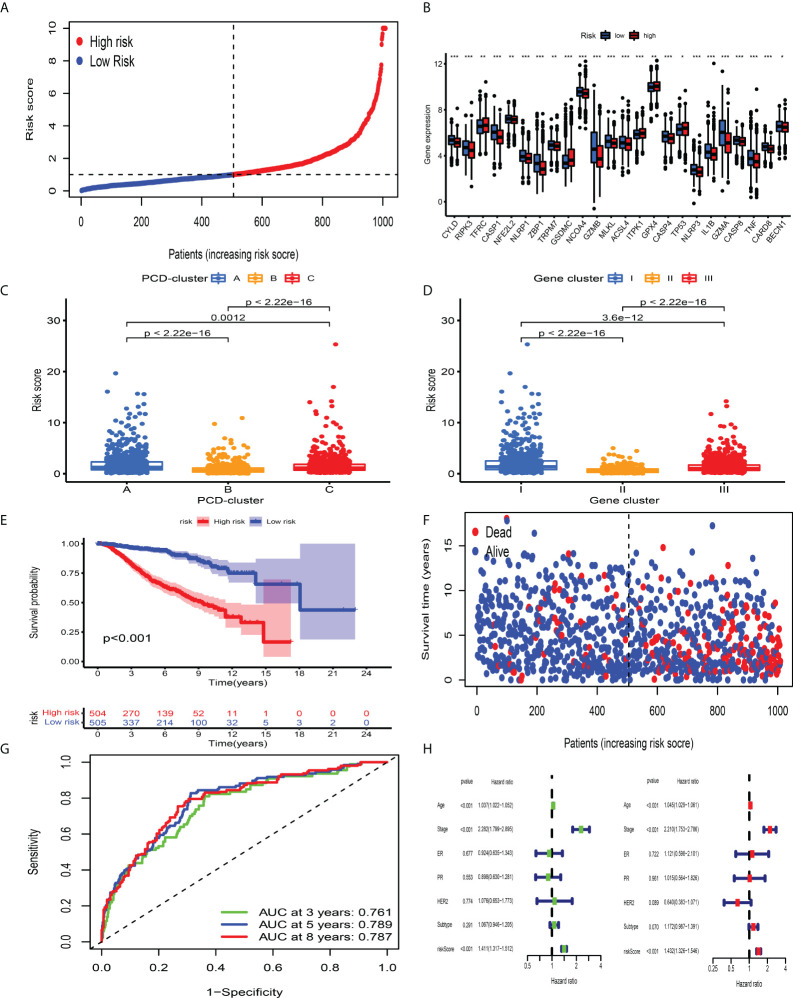
Survival analysis of CD_Score in the development dataset. **(A)** Ranked dots showing the risk score distribution. **(B)** Difference in the expression level of PCDAGs between high and low risk-score group. The asterisks represented the statistical P-value (*P < 0.05; **P < 0.01; ***P < 0.001). **(C, D)** Distribution of risk score in the different PCD-clusters and gene clusters. **(E)** Kaplan–Meier curves for the OS of patients between high and low risk-score group. **(F)** Scatter plots showing the risk score distribution and patient survival status. **(G)** ROC curves to predict the sensitivity and specificity of 3-, 5-, and 8-year survival according to CD_Score in the discovery dataset. **(H)** Results of the univariate and multivariate Cox regression analyses regarding OS in the meta-cohort.

Although BC patients could be classified into groups with distinct prognosis and antitumor immunity by consensus clustering analysis, a PCD-related scoring model to evaluate the possibility of OS has not been hitherto identified for BC clinicians. The meta-cohort, including 2,038 BC patients, was randomized into the discovery and validation datasets, both of which contained 1,009 samples (at a ratio of 1:1). In the discovery dataset, we employed the LASSO regression algorithm to further screen prognostic PCD-related signature genes. According to the minimum partial likelihood deviance and optimum λ value (**Figures S7A, B**), we identified 41 genes with prognostic value and incorporated them into the multivariate Cox regression model. Based on the optimum AIC value, an eighteen-gene scoring system was constructed, whose formula is presented in **Table S13**. To explore the protein expression patterns of these eighteen genes, we performed immunohistochemistry analysis and discovered that, with the exception of C15orf39, HSD11B1, LIMD2, and low/medium/high protein expressions of other genes were observed in BC tissues (**Figure S8**). According to the risk score obtained from the formula, we classified BC patients into CD_Score-low and -high group (**Figure 5A**) and discovered an obvious difference in PCDAG expression between different groups (**Figure 5B**). Besides, we observed that the distribution of CD_Score differed from distinct PCD-clusters and gene clusters, among which PCD-cluster A and gene cluster I with dismal survival outcomes were highly correlated to high CD_Score (**Figures 5C, D**). As expected, further survival analysis and risk score distribution demonstrated that compared with the CD_Score-low group, the CD_Score-high group exhibited a relatively poor outcome and had a higher likelihood of death earlier (**Figures 5E, F**). Besides, the AUC value was utilized to represent the 3-, 5-, and 8-year survival rates of CD_Score: 0.761, 0.789, and 0.787, respectively (**Figure 5G**). Uni- and multi-variate analyses were conducted to determine the independent role of CD_Score in predicting BC prognosis (**Figure 5H**).

The validation dataset was used to evaluate the performance of CD_Score. By calculating the risk score of each BC sample premised on the same formula derived from the discovery dataset, we divided them into CD_Score-low and -high group, with the median value considered as the cutoff level (**Figure S7C**). CD_Score-low BC patients were less likely to encounter death earlier and exhibited favorable outcome compared with those CD_Score-high counterparts (**Figures S7D, E**). The AUC of 3-, 5-, and 8-year ROC for the CD_Score in the validation dataset was 0.741, 0.724, and 0.695, respectively (**Figure S7F**).

### Correlation between CD_Score and clinicopathological characteristics and molecular subtypes

Taking into consideration the heterogeneous properties of BC, we also evaluated the clinical value of CD_Score in predicting prognosis among different subgroups (age, molecular subtype, clinical stage, hormone receptor, and HER2 status) and further confirmed the role of CD_Score as a robust prognostic biomarker (**Figure S9**). Besides, the distribution of CD_Score in different groups with ER, PR, HER2 status, molecular subtype and clinical stage was also compared, and we observed that low CD_Score was linked with ER+, PR+ status, while high CD_Score correlated with HER2+ status, basal and Her2 subtype and stages iii and iv (**Figure S10**). To further assess the predictive performance of CD_Score, we conducted a comparative analysis between this scoring system and other established gene expression signatures from the perspective of autophagy, ferroptosis, pyroptosis, and m6A modification. Kaplan–Meier analysis combined with ROC curve demonstrated that CD_Score based on a combination of ferroptosis, necroptosis, and pyroptosis was superior to that based on individual cell death modality and m6A modification, in terms of predicting survival outcome for BC patients (**Figure S11**).

### TME-infiltrating immune landscape and clinical nomogram associated with CD_Score

To explore the underlying mechanisms contributing to the survival discrepancy between different CD_Score groups, we conducted GSEA and observed that immune-related biological processes were markedly overrepresented in the CD_Score-low group (**Figure S12A**). The specific association between the CD_Score and immune characterization was investigated through Spearman analysis and illustrated as the correlation matrix (**Figure S12B**). We observed that CD_Score exhibited a positive correlation with immunosuppressive immune cells, including Treg cells and M2 macrophages, and displayed a negative association with tumor-infiltrating lymphocytes, especially CD8^+^ T cells, highlighting the interaction between CD_Score and immune infiltration characteristics. By integrating the CD_Score and other clinicopathological characteristics, a clinical nomogram was finally constructed, which exhibited sense discrimination and accurate calibration (**Figures S12C–E**).

### Correlation between CD_Score and somatic mutation and response to immunotherapies and chemotherapeutics

**Figure 6:**
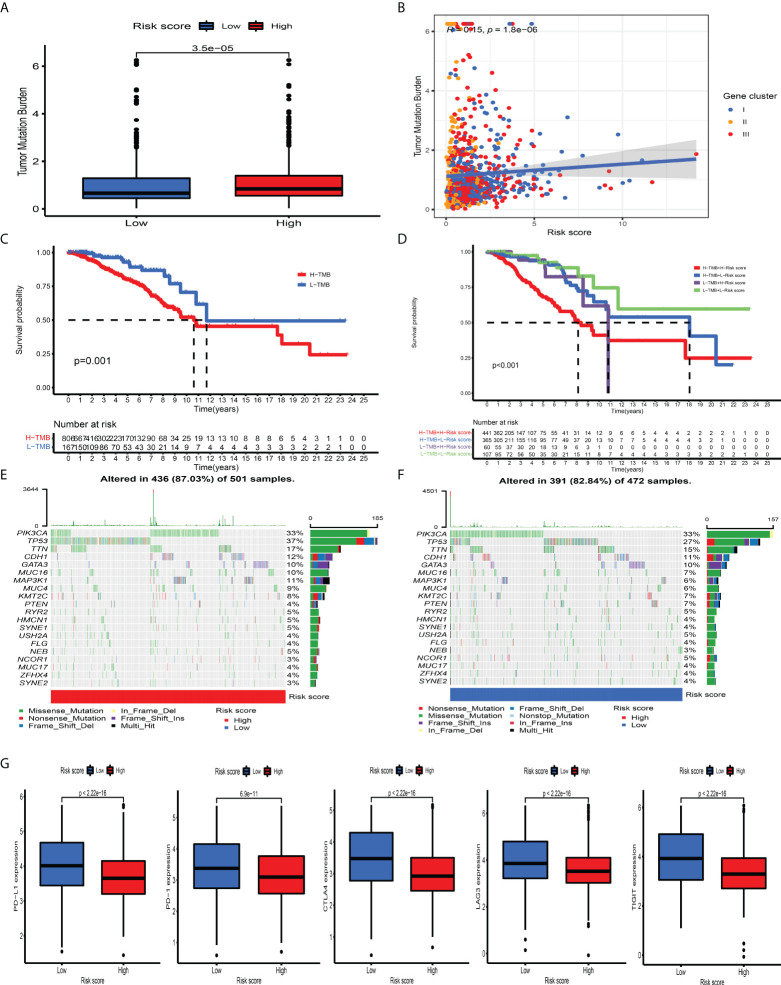
The association between tumor mutation burden and immunotherapeutic benefits. **(A)** Relative distribution of TMB in high versus low risk-score group. **(B)** correlation analysis between risk score and TMB. **(C)** Kaplan–Meier curves for high and low TMB patient groups. **(D)** Kaplan–Meier curves for subgroup patients stratified by both risk score and TMB. **(E, F)** Mutational landscape of SMGs in the TCGA-BRCA stratified by high (left panel) versus low risk-score (right panel) groups. Individual patients were represented in each column. The upper bar plot showed TMB, the right bar plot showed the mutation frequency of each gene in separate risk-score groups. **(G)** The relative distribution of immunosuppressive molecules was compared between risk score high versus low groups in the TCGA-BRCA dataset.

Mounting studies have confirmed that TMB, as an emerging and promising tumor marker, could assist us in selecting patients suitable for immunotherapies. Taking into consideration the significant value of TMB in clinical practice, we aimed to uncover the distribution pattern of TMB between different CD_Score groups and clarify the genetic imprint of each group. As shown in **Figures 6A, B**, CD_Score exhibited a positive correlation with TMB and CD_Score-high patients were mainly enriched in the high TMB group. Subsequent analysis demonstrated the prognostic impact of TMB by categorizing BC patients into TMB-low and -high group with 0.421 as the cutoff value. We discovered that the TMB-high group displayed a dismal OS disadvantage and—especially the combined TMB-high and CD_Score-high group—demonstrated the worst survival outcome (**Figures 6C, D**). The SMGs landscape between different CD_Score groups was also analyzed and we discovered that TP53 (36.93% vs. 26.69%) and MAP3K1 (10.58% vs. 5.72%) displayed a relatively high somatic mutation frequency in the CD_Score-high group (**Figures 6E, F**; **Table 1**). Besides, to explore the clinical relevance of TP53 and MAP3K1 expression, we analyzed their protein expression in clinical specimens. According to the immunohistochemical analyses, we discovered that TP53 and MAP3K1 demonstrated moderate and high staining intensities in BC tissues, respectively (**Figures S13A, B**). Moreover, TP53 and MAP3K1-related signaling pathways based on GSEA were used to explore signaling pathways involved in BC between high and low expression datasets. We discovered that KEGG items, including cell cycle and spliceosome, were significantly enriched in the TP53 high expression phenotype, while oxidative phosphorylation and ribosome demonstrated significantly differential enrichment in the MAP3K1 low expression phenotype (**Figures S13C, D**). Considering that tumor-infiltrating lymphocytes could influence tumor progression and survival outcome, we further explored the correlation between TP53, MAP3K1 and TME landscape in BC patients. As shown in **Figures S13E, F**, we discovered that B cells naïve NK cells activated and macrophages M2 were the main immune cells affected by TP53, while T cells CD4 memory activated, T cells follicular helper and T cells regulatory demonstrated a negative correlation with MAP3K1.

**Table 1:** Significantly mutated genes between CD_Score-low and -high group.

gene	H-wild	H-mutation	L-wild	L-mutation	P-value
**TP53**	316 (63.07%)	185 (36.93%)	346 (73.31%)	126 (26.69%)	<0.001
**MAP3K1**	448 (89.42%)	53 (10.58%)	445 (94.28%)	27 (5.72%)	<0.05

H, high CD_Score group; L, low CD_Score group.

ICI-mediated immunotherapy, especially PD-1/CTLA-4 (extensively used to evaluate immune response), has gained remarkable success in antitumor immunity. By comparison with the expression level of ICI between the CD_Score-low and -high group, we discovered that PD-1, PD-L1, LAG3, CTLA4, and TIGIT were markedly increased in the CD_Score-low group (**Figure 6G**). Besides, we discovered that no matter the circumstances of anti-CTLA4/PD-1 therapy alone or in combination, the CD_Score-low group exhibited a relatively higher IPS score, compared with the CD_Score-high group (**Figure S14A**). These findings strongly suggested that CD_Score performed an indispensable role in the mediation of immune response and the prediction of immunotherapeutic responsiveness.

Besides, the difference in drug IC_50_ between the CD_Score-low and -high group was compared, and we discovered that CD_Score-low group demonstrated a negative correlation with the IC50 of gemcitabine, doxorubicin, cisplatin, methotrexate, vinorelbine, and vinblastine, suggesting that these commonly used chemotherapeutic agents could confer beneficial effects on them (**Figure S14B**).

## Discussion

An accumulating body of evidence has highlighted that ferroptosis, pyroptosis, and necroptosis perform a vital role in the regulation of inflammation response and antitumor activity (54–56). Numerous studies have explored the significant contribution of individual cell death modalities, but comprehensive analysis of the crosstalk of distinct cell death types in tumor progression has not been performed. Here, we revealed global alterations of PCDAGs from the perspective of genomics and transcriptomics, and explored the crosstalk between distinct cell death modalities and tumor-infiltrating immune cells to determine distinct PCD-mediated patterns in the immune landscape of TME. This would advance our understanding of antitumor immunity and facilitate the implementation of more effective immunotherapeutic approaches.

In this research, we determined three distinct PCD-mediated tumor patterns, which exhibited different TME-infiltrating immune cell landscapes and anti-tumor immunity. PCD-cluster B was characterized by abundant tumor-infiltrating lymphocytes and immune-activating components, corresponding to an “inflamed” immune profile. PCD-cluster A was characterized by higher tumor impurity and immune-suppressive TME, characterized by a “desert” immune profile. PCD-cluster C was featured by the immune cell infiltration together with activated ECM, TGF beta, and EMT pathways, corresponding to an “excluded” immune profile. Previous studies have established that TME constituents take on an indispensable role in regulating cancer progression and influencing immunotherapeutic efficacy (24). Baseline characteristics of immune cell contextures, including NK cells, macrophage M1 and CD4/CD8^+^ T cells significantly correlated with immune response (57, 58). We discovered that anti-tumor infiltrating lymphocytes and immunosuppressive molecules were prominently overrepresented in PCD-cluster B, highlighting the potential value of the crosstalk of distinct cell death modalities in predicting the efficacy of immunotherapies. Recently, numerous studies identified that activated TGF beta and EMT-related pathways could restrict the penetration of lymphocyte subpopulations into the tumor parenchyma (59), while targeted small molecule (TGF-β) inhibitor therapy could restore antitumor immunity *via* remodeling the TME feature (60, 61). These findings implied that BC patients within PCD-cluster C were the potential candidates receiving therapeutic benefits from combination of TGF-β blockade and ICI immunotherapy.

Furthermore, DEGs extracted from distinct PCD-clusters were significantly enriched in biological activity associated with anti-tumor immunity, demonstrating that these DEGs were recognized as PCD-related gene signature. Based on the PCD-related signature genes, three PCD-related transcriptome subtypes characterized by distinct clinical outcomes and TME features were established, which was consistent with the results obtained from PCD-clusters. We further introduced the CD_Score to estimate the survival ability and predict therapeutic efficacy for BC patients. Consequently, PCD-cluster B, characterized by an “inflamed” immune profile, exhibited a lower CD_Score, while cluster C and A, characterized by an “excluded” and “desert” immune profile, displayed a relatively high CD_Score. Additionally, we discovered that CD_Score possessed great prognostic predictive ability and exhibited a significant correlation with mutational signatures, demonstrating that CD_Score could be used as a surrogate biomarker to predict genomic aberration. Further analyses revealed that CD_Score exhibited a significant correlation with immune response, including immunosuppressive molecules, IPS, and TME landscape, demonstrating that PCD could affect the efficacy of immunotherapeutic approaches. Taken together, we believed that CD_Score could be utilized in clinical practice to determine immune phenotypes and guide therapeutic approaches.

Due to cancer being a genetic disease, the detection of mutation driver genes could be helpful in monitoring cancer occurrence and determining treatment options. Here, we discovered that TP53 and MAP3K1 displayed an increased mutation frequency in the CD_Score-high group. Previous studies reported that MAP3K1, as a serine-threonine kinase of the MAPK family, is frequently mutated in breast cancer and further influences Th1 polarization, leading to an immune-desert phenotype in BC (62). TP53 is a frequently mutated tumor suppressor gene in BC, and its mutation could strengthen immune function (63). The CD_Score mediated driver gene mutations exhibited a significant correlation with immune activity, suggesting the complicated interplay between PCD and tumor immunogenomic characteristics.

Although we incorporated 34 identified PCDs from the GeneCards database, novel identified regulators will be recognized and incorporated into this research to enhance the accuracy of PCD-mediated patterns. Besides, we established CD_Score based on the retrospective meta-cohort containing 2,038 BC samples. Prospective cohort studies were warranted to further validate the applicability of our findings.

## Conclusions

In general, based on 34 recognized PCDAGs, we systematically explored PCD-mediated patterns and CD_Score among 2,038 BC samples and integrated these patterns with the TME-infiltrating landscape. Through the comprehensive analysis, we concluded that the crosstalk of ferroptosis, pyroptosis, and necroptosis played a crucial role in regulating antitumor immunity. More broadly, evaluation of the CD_score of BC patients will help strengthen our understanding of the immune landscape of TME and direct more effective clinical practice of immunotherapies.

## Data availability statement

The datasets presented in this study can be found in online repositories. The names of the repository/repositories and accession number(s) can be found in the article/**Supplementary Material**.

## Author contributions

LX conceived and designed the study, provided analytical technical support, drafted the manuscript and participated in the production of charts and pictures. The author has made a substantial, direct, and intellectual contribution to the work and approved it for publication.

## Conflict of interest

The authors declares that the research was conducted in the absence of any commercial or financial relationships that could be construed as a potential conflict of interest.

## Publisher’s note

All claims expressed in this article are solely those of the authors and do not necessarily represent those of their affiliated organizations, or those of the publisher, the editors and the reviewers. Any product that may be evaluated in this article, or claim that may be made by its manufacturer, is not guaranteed or endorsed by the publisher.

## Abbreviations

BC, breast cancer; ICI, immune checkpoint inhibitor; OS, overall survival; PCD, programmed cell death; TME, tumor microenvironment; CNV, copy number variation; TMB, tumor mutation burden; PCDAGs, programmed cell death associated genes; GSVA, gene set variation analysis; ssGSEA, single-sample gene set enrichment analysis; ESTIMATE, Estimation of Stromal and Immune Cells in Malignant Tumors using Expression Data; DEGs, differentially expressed genes; SMGs, significantly mutated genes; IPS, immunophenoscore; IC_50_, the half inhibitory concentration.
