# Supplementary material for: Crosstalk of three novel types of programmed cell death defines distinct microenvironment characterization and pharmacogenomic landscape in breast cancer
Source: Front Immunol. 2022 Aug 11;13:942765. doi: 10.3389/fimmu.2022.942765 (PMC9403178; doi:10.3389/fimmu.2022.942765)
Supplement: Supplementary file 1 [file DataSheet_1.docx]

**SUPPLEMENTARY FIGURES S1-S14**

**
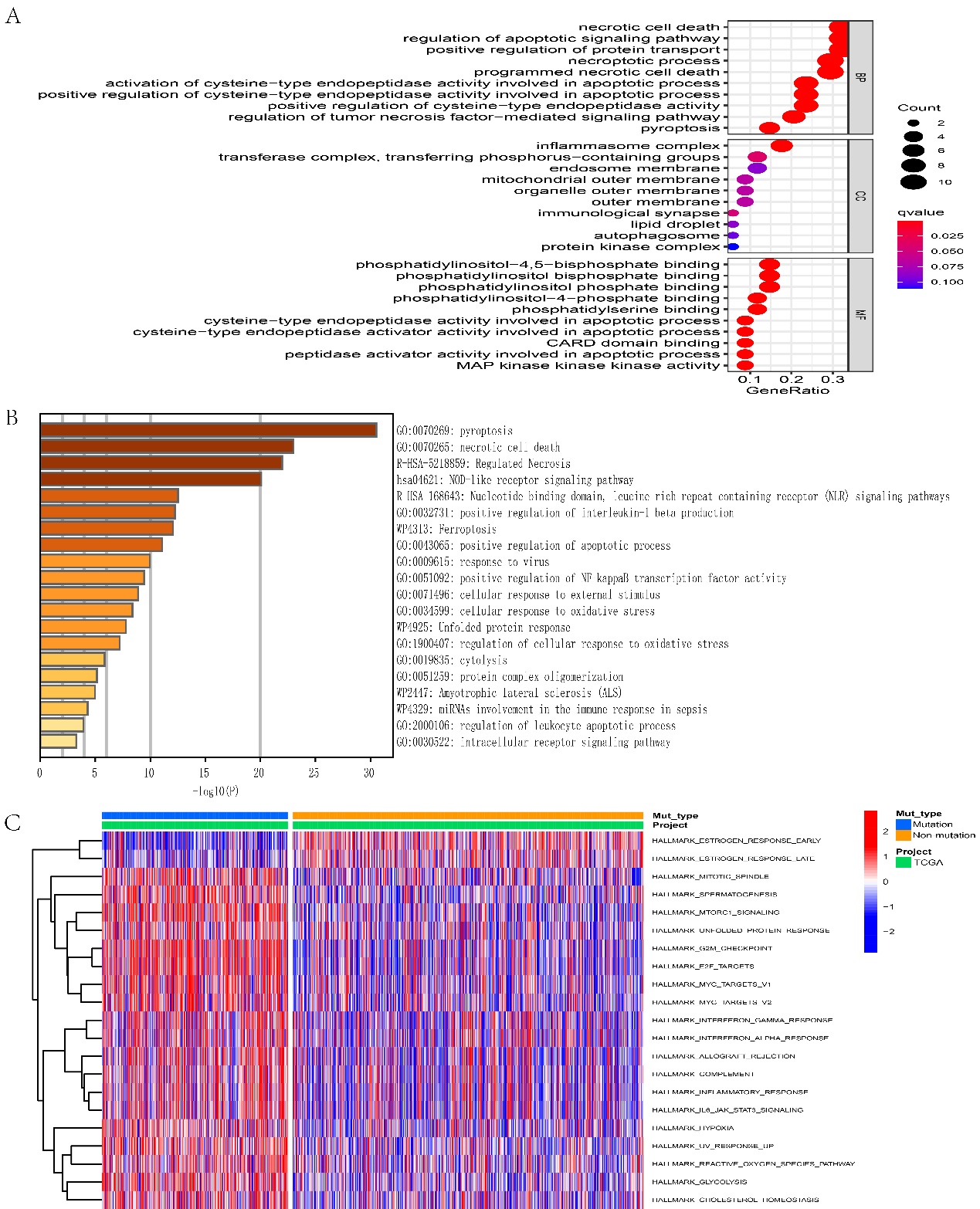
**

**Figure S1. Functional annotation and mutation characteristics of programmed cell death-associated genes in the TCGA-BRCA dataset. (A, B)** GO and Metascape enrichment analysis of PCDAGs. **(C)** GSVA enrichment analysis displayed the differences of biological processes between PCDAGs mutation and non-mutation tumors.

**
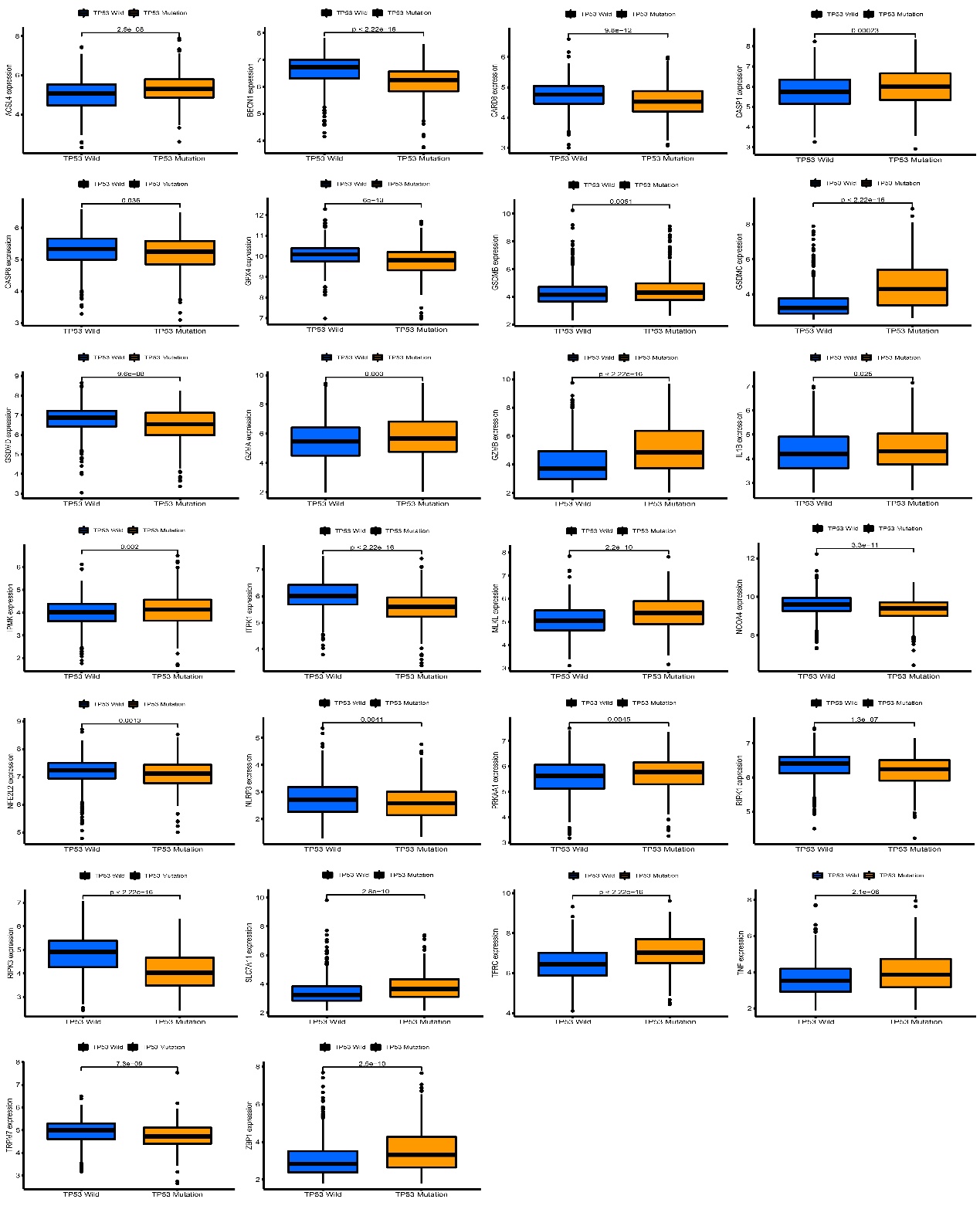
**

**Figure S2. The relationship between the expression level of programmed cell death-associated genes and TP53 mutation in breast cancer.**

**
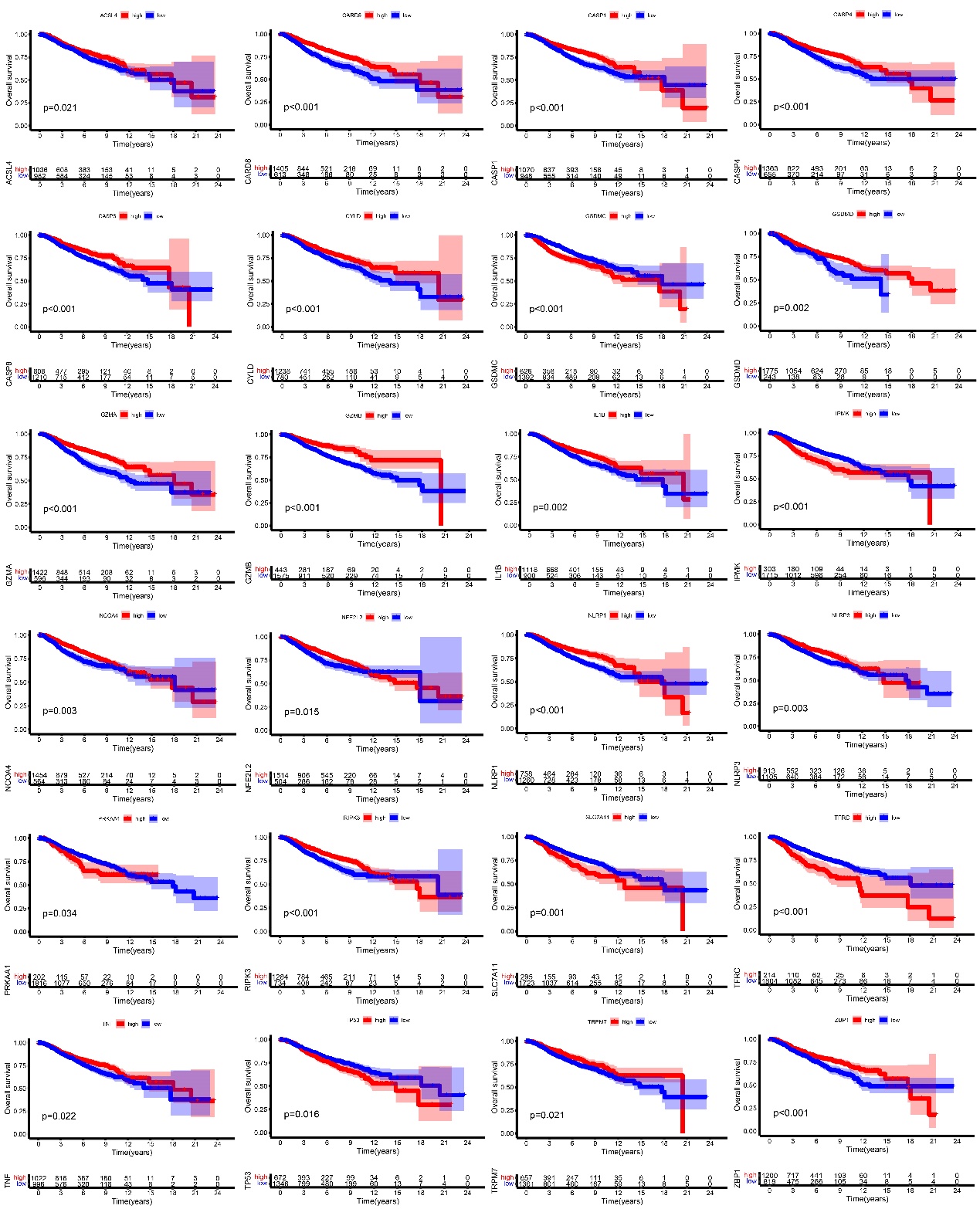
**

**Figure S3. The prognostic impact of programmed cell death-associated genes in breast cancer by univariate Cox regression and Kaplan-Meier analysis.**

**
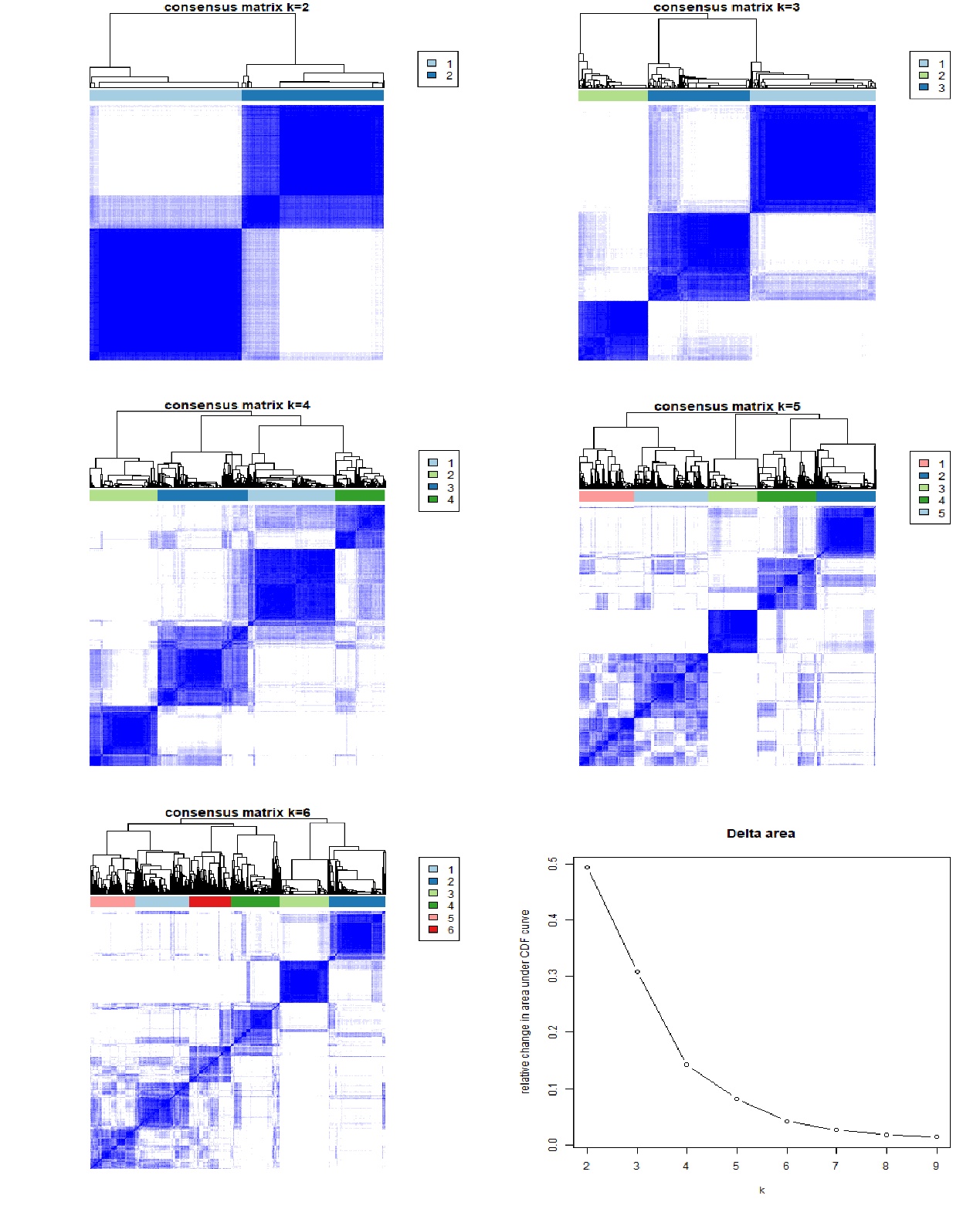
**

**Figure S4. Unsupervised clustering of programmed cell death-associated genes and Consensus matrix heatmaps for k= 2-6.**

**
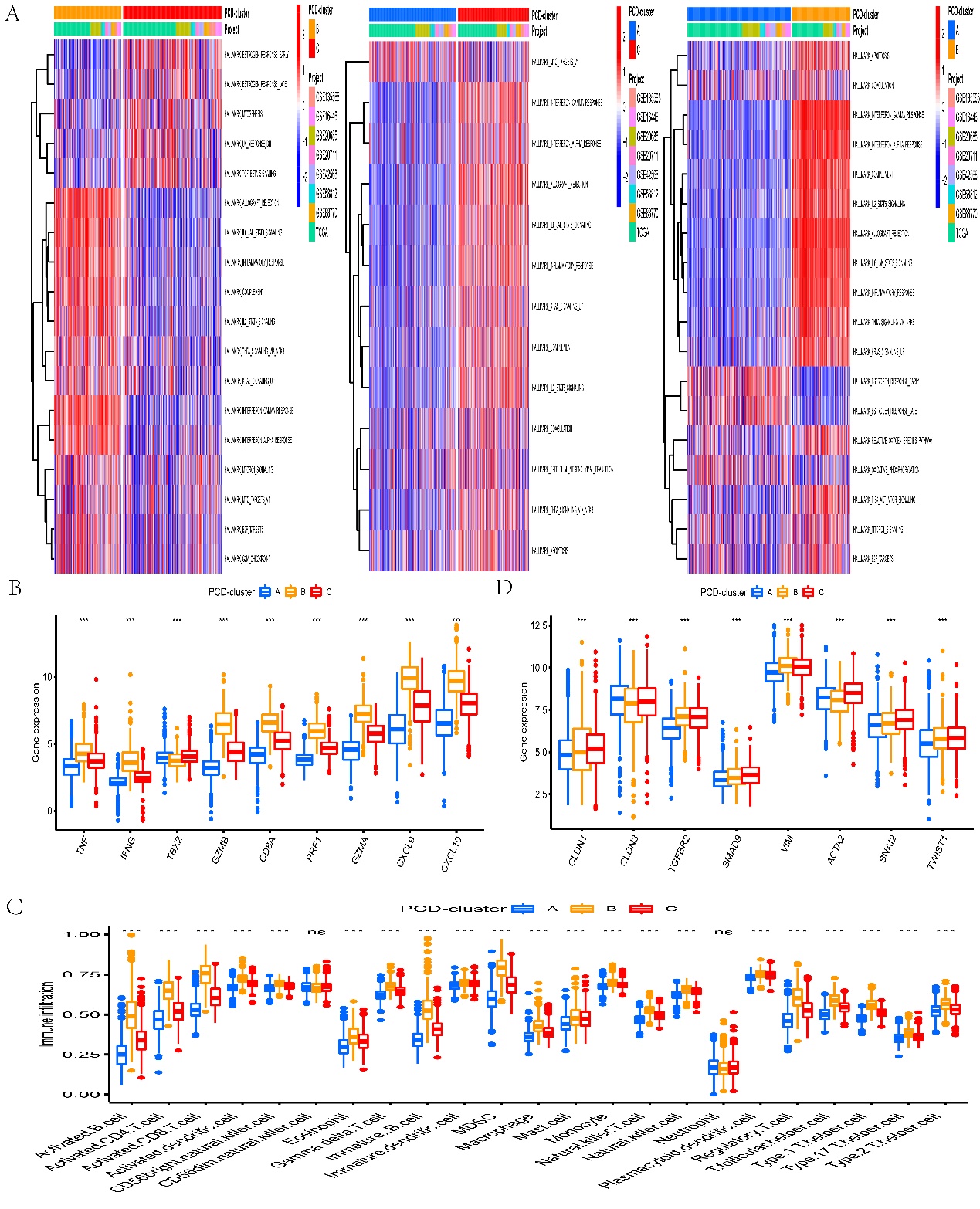
**

**Figure S5. Biological behaviors and tumor microenvironment characteristics among distinct PCD-clusters. (A)** Heatmap shows the GSVA score of representative Hallmark pathways curated from MSigDB among distinct PCD-mediated tumor patterns. **(B)** Comparison of the expression level of immune activation-related transcripts across distinct PCD-clusters. **(C)** The fraction of antitumor lymphocyte subpopulations among distinct PCD-clusters using the ssGSEA algorithm. **(D)** Comparison of the expression level of transcripts of TGF beta/EMT pathway across distinct PCD-clusters.

**
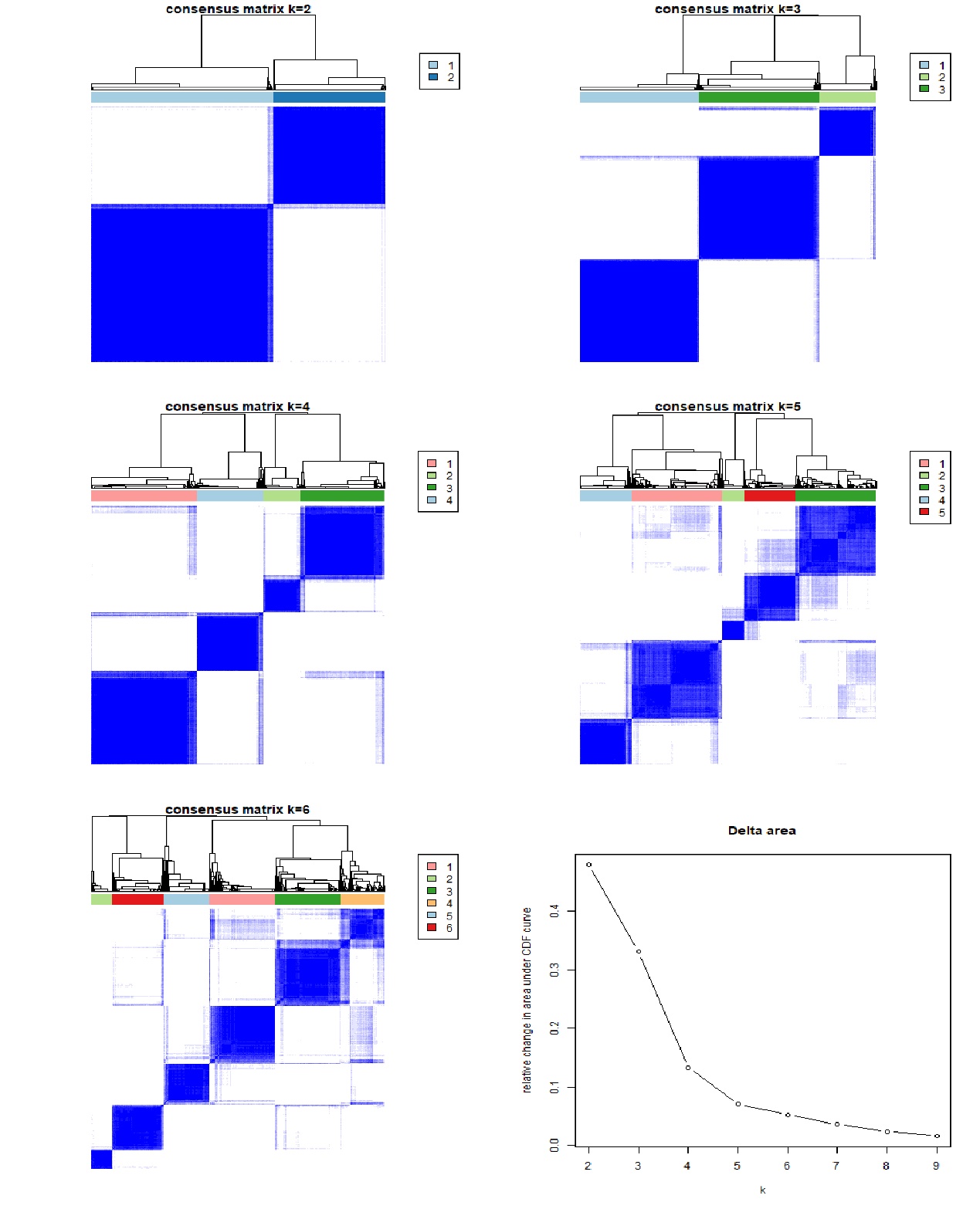
**

**Figure S6. Unsupervised clustering of prognostic programmed cell death-related signature genes and Consensus matrix heatmaps for k= 2-6.**

**
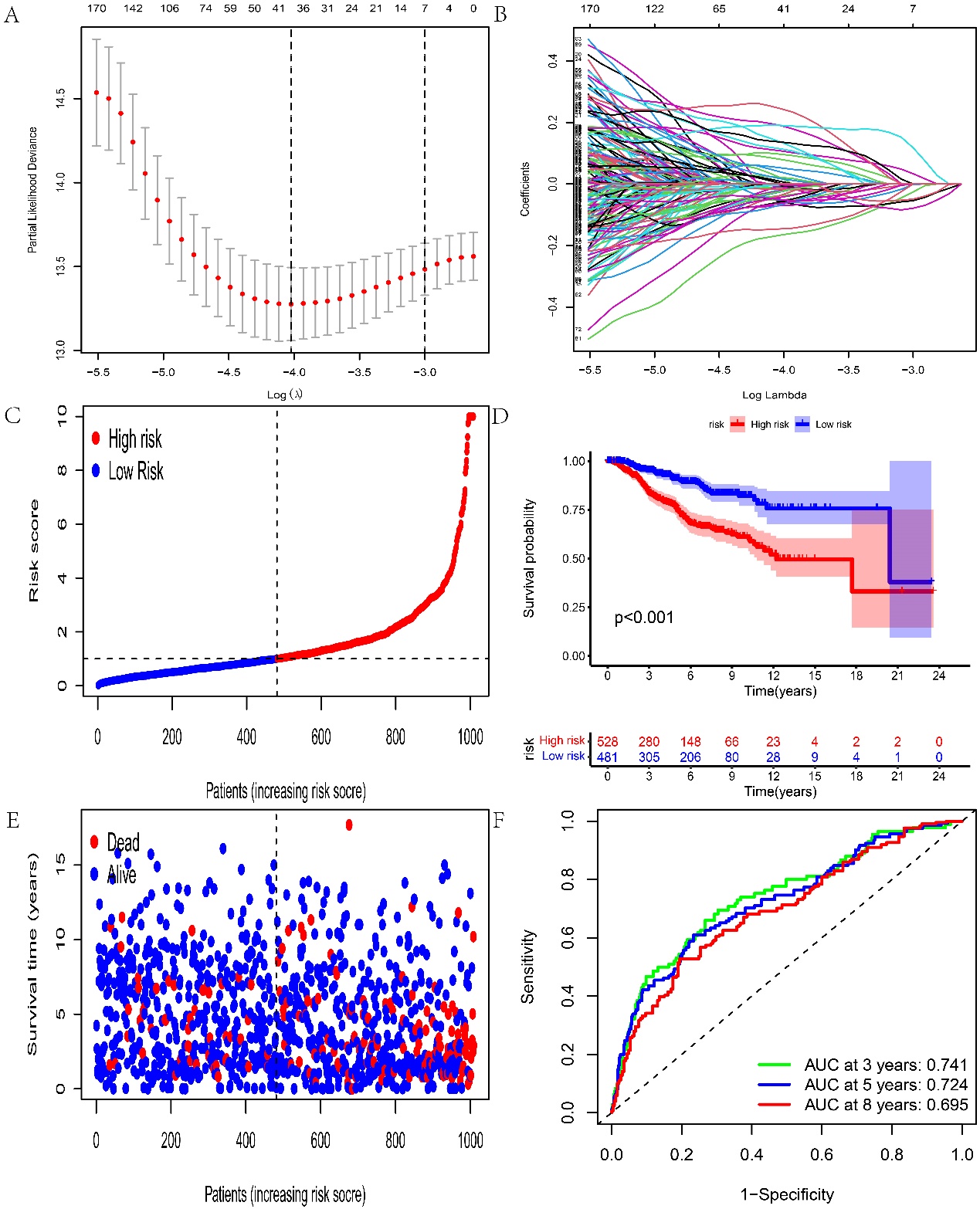
**

**Figure S7. Survival analysis of CD_Score in the validation dataset. (A, B)** The LASSO regression analysis and partial likelihood deviance on the prognostic PCD-related signature genes. **(C)** Ranked dots showing the risk score distribution. **(D)** Kaplan-Meier curves for the OS of patients between high and low risk-score group. **(E)** Scatter plots showing the risk score distribution and patient survival status. **(F)** ROC curves to predict the sensitivity and specificity of 3-, 5- and 8-year survival according to CD_Score in the validation dataset.

**
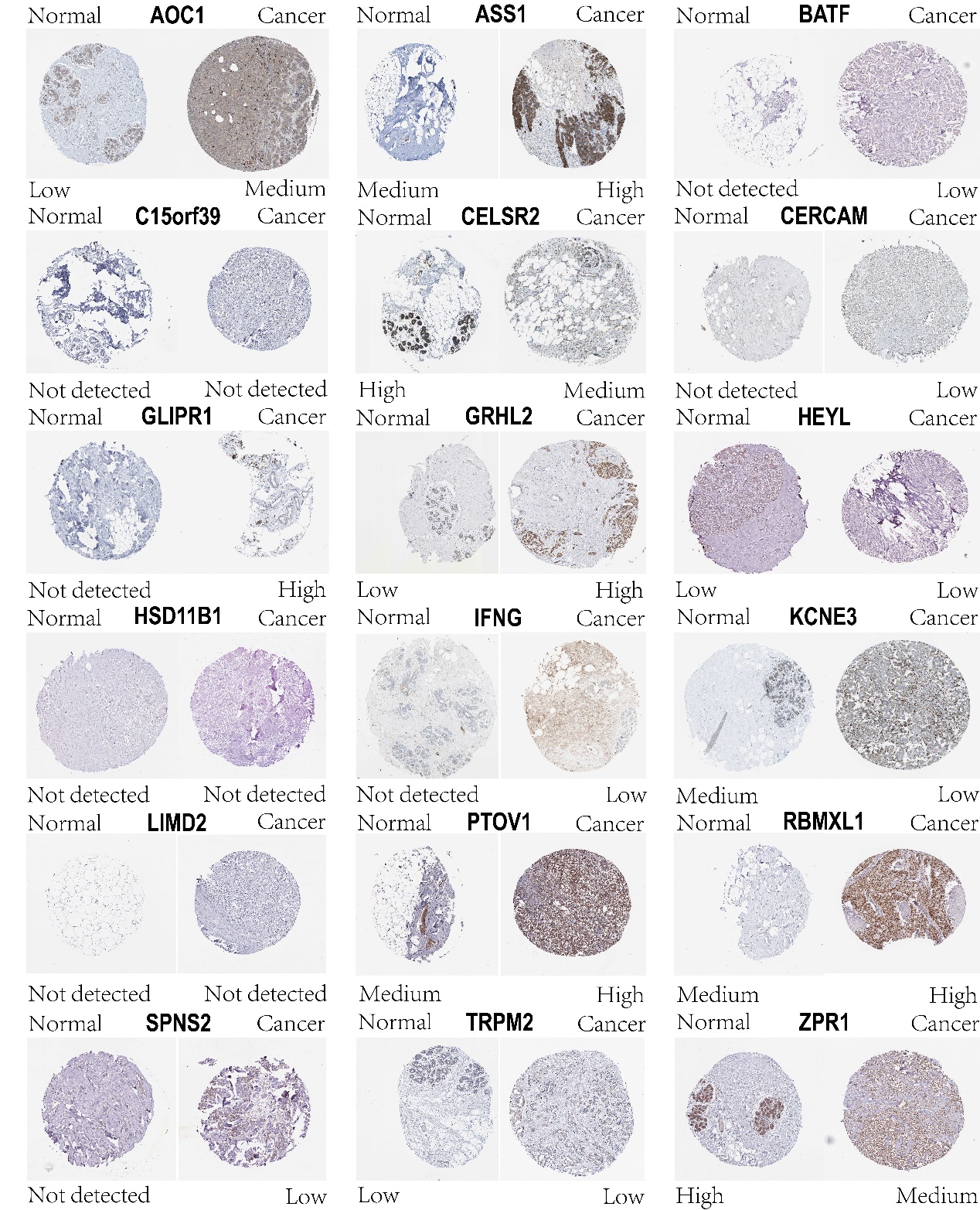
**

**Figure S8. Representative immunohistochemistry images of eighteen genes in breast cancer and normal tissues via Human Protein Atlas.**

**
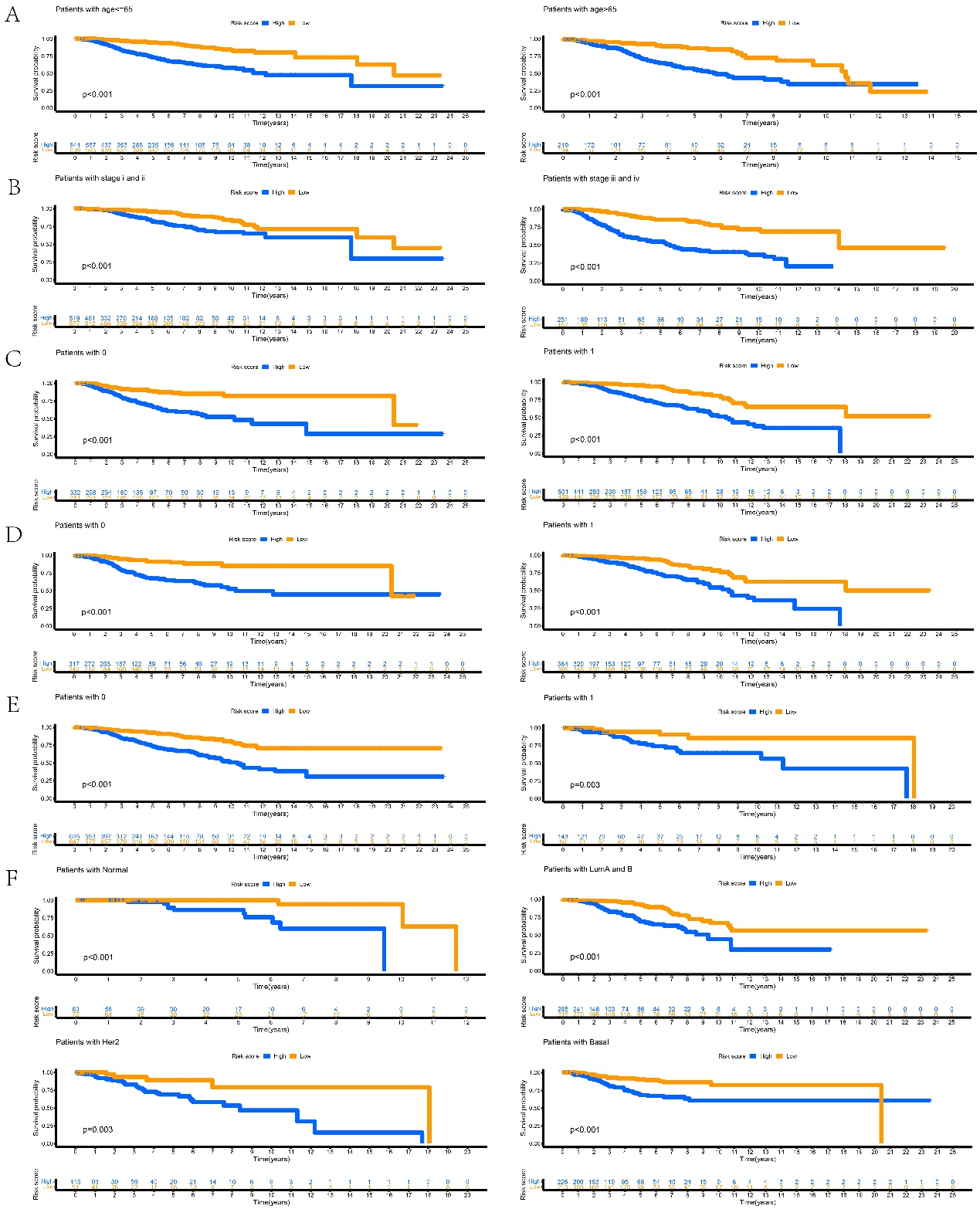
**

**Figure S9. Subgroup analysis of the prognostic role of CD_Score in clinicopathological characteristics, including age, ER, PR, HER2 status, molecular subtype and clinical stage.**

**
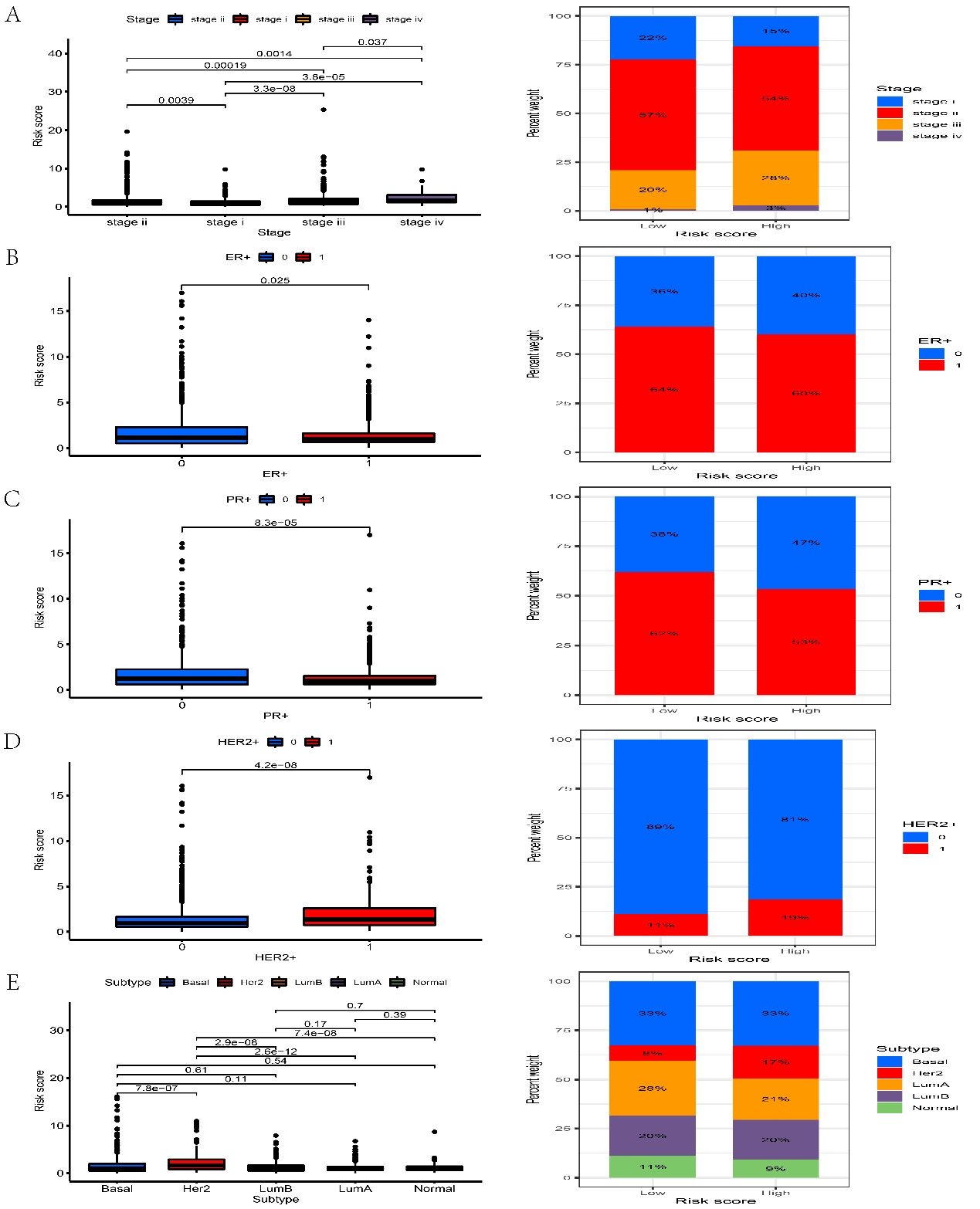
**

**Figure S10. The distribution of CD_Score in groups with clinicopathological characteristics, including clinical stage, ER, PR, HER2 status and molecular subtype.**


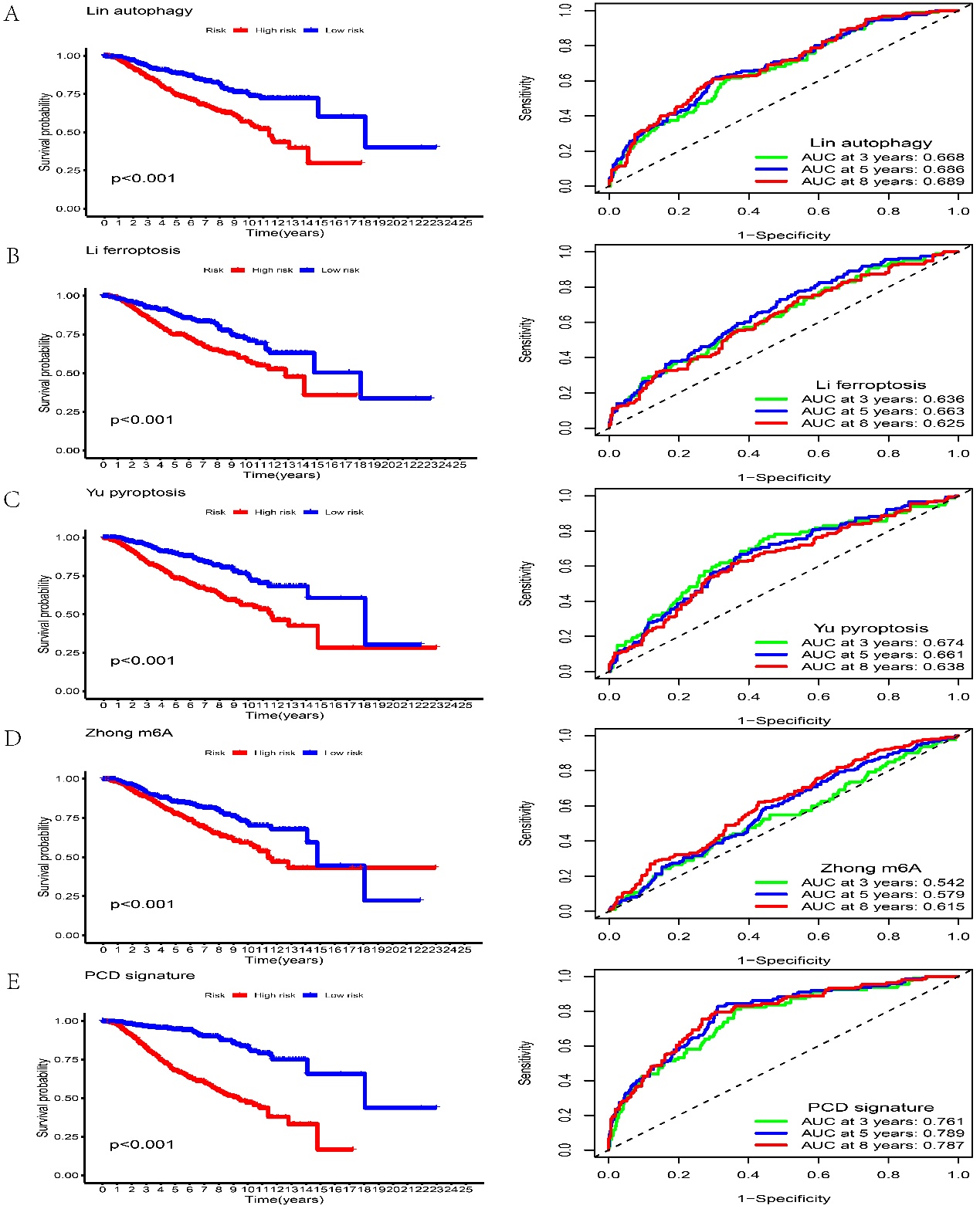


**Figure S11. Comparison between CD_Score and other established gene expression signature from the perspective of autophagy ferroptosis, pyroptosis, and m6A modification in breast cancer.**


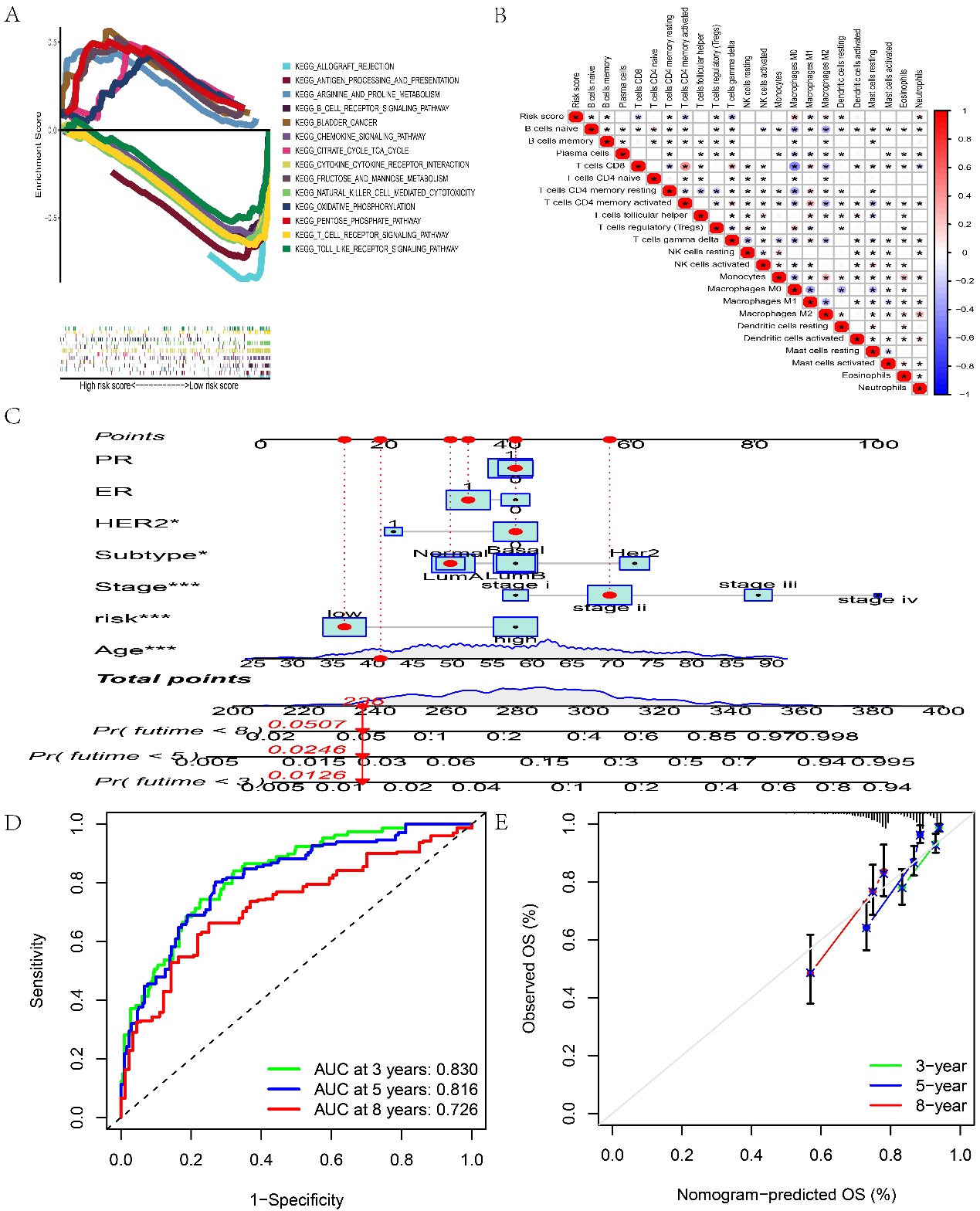


**Figure S12. Tumor microenvironment characteristics and clinical nomogram. (A)** Representative results of KEGG pathways between high and low risk-score group via GSEA. **(B)** The association between CD_Score and TME-infiltrating immune cells using Spearman analysis. **(C)** Nomogram based on CD_Score and clinicopathological characteristics including age, ER, PR, HER2 status, molecular subtype and clinical stage. **(D)** ROC curves to predict the sensitivity and specificity of 3-, 5- and 8-year survival according to clinical nomogram. **(E)** Calibration curves of the nomogram for OS prediction at 3, 5 and 8 years for BC patients.

**
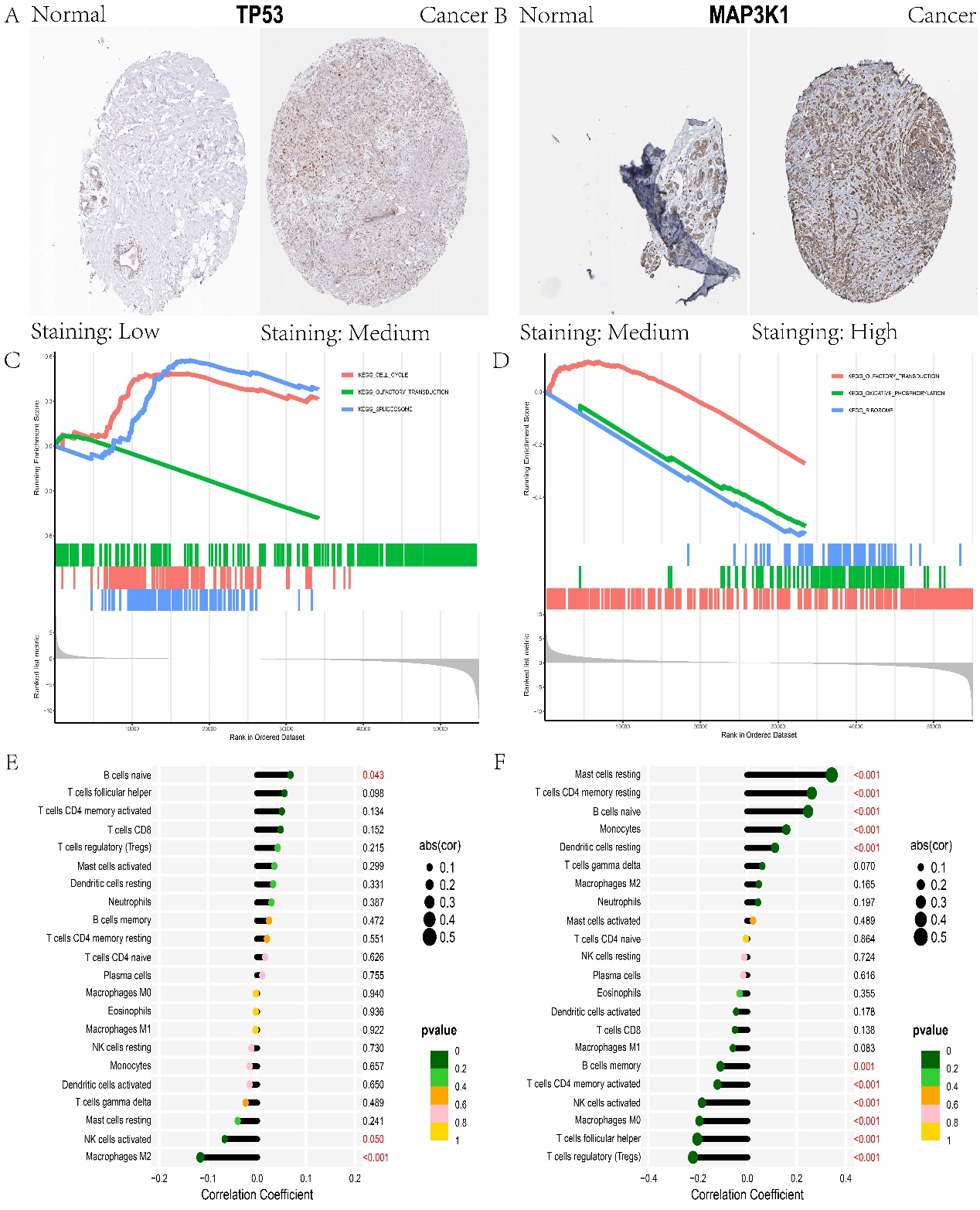
**

**Figure S13. Expression and biological behaviors of TP53 and MAP3K1 throughout breast cancer tissues. (A, B)** Protein expression of TP53 and MAP3K1 in BC specimens via the Human Protein Atlas. **(C, D)** Representative results of KEGG pathways for TP53 and MAP3K1 via GSEA. **(E, F)** Correlation analysis between TP53, MAP3K1 and TME landscape.

**
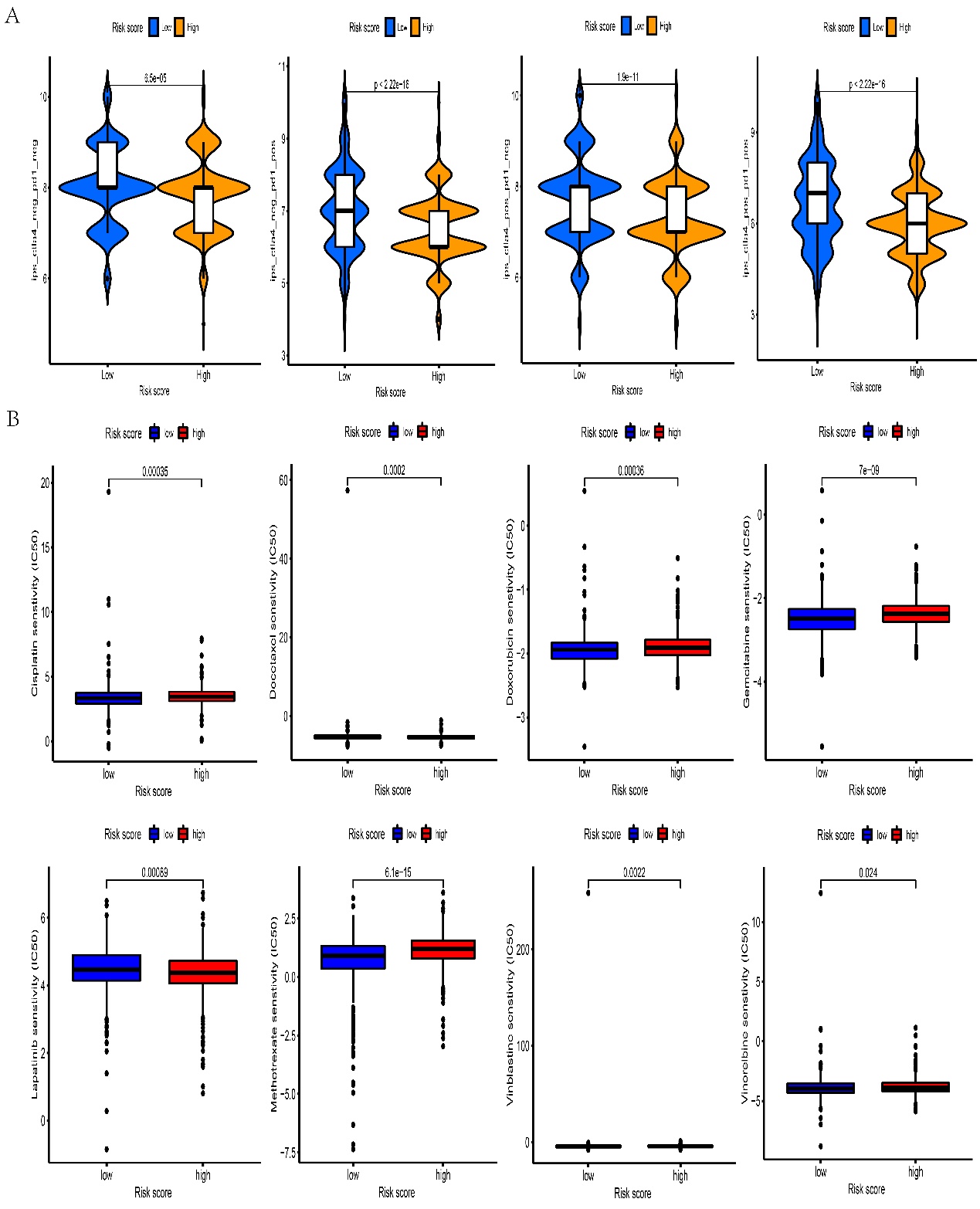
**

**Figure S14. Estimation of the role of CD_Score in immunotherapeutic efficacy and drug susceptibility.**
